# Independent Mechanisms Lead to Genomic Instability in Hodgkin Lymphoma: Microsatellite or Chromosomal Instability †

**DOI:** 10.3390/cancers10070233

**Published:** 2018-07-13

**Authors:** Corina Cuceu, Bruno Colicchio, Eric Jeandidier, Steffen Junker, François Plassa, Grace Shim, Justyna Mika, Monika Frenzel, Mustafa AL Jawhari, William M. Hempel, Grainne O’Brien, Aude Lenain, Luc Morat, Theodore Girinsky, Alain Dieterlen, Joanna Polanska, Christophe Badie, Patrice Carde, Radhia M’Kacher

**Affiliations:** 1 Radiobiology and Oncology Laboratory, CEA, iRCM, 92265 Fontenay aux Roses CEDEX, France; cuceu_corina@yahoo.com (C.C.); graceshim1@gmail.com (G.S.); monika.frenzel@hotmail.com (M.F.); mustafa.aljawhari@hotmail.fr (M.A.J.); williamhempel824@gmail.com (W.M.H.); audelenain@yahoo.fr (A.L.); luc.morat@cea.fr (L.M.); 2 IRIMAS, Institut de Recherche en Informatique, Mathématiques, Automatique et Signal, Université de Haute-Alsace, 68093 Mulhouse, France; bruno.colicchio@uha.fr (B.C.); alain.dieterlen@uha.fr (A.D.); 3 Department of Genetic, Groupe Hospitalier de la Région de Mulhouse Sud-Alsace, 68093 Mulhouse, France; jeandidiere@ghrmsa.fr; 4 Institute of Biomedicine, University of Aarhus, DK-8000 Aarhus, Denmark; sjunker@biomed.au.dk; 5 Laboratory of Biochemistry B, Saint Louis Hospital, 75010 Paris, France; lfplassa@yahoo.fr; 6 Faculty of Automatic Control, Electronics and Computer Science, Silesian University of Techology, 44-100 Gliwice, Poland; justyna.mika@polsl.pl (J.M.); Joanna.Polanska@polsl.pl (J.P.); 7 Biological Effects Department, Centre for Radiation, Chemical and Environmental Hazards, Public Health England, Didcot OX11 ORQ, UK; Grainne.o'brien@phe.gov.uk (G.O.); christophe.badie@phe.gov.uk (C.B.); 8 Department of Radiation Oncology, Gustave Roussy Cancer Campus, 94805 Villejuif, France; theogirinsky@me.com; 9 Department of Medicine, Gustave Roussy Cancer Campus, University Paris Saclay, 94805 Villejuif, France; dr.pcarde@gmail.com; 10 Cell Environment DNA Damages R&D Oncology Section, 75020 Paris, France

**Keywords:** Hodgkin lymphoma, chromosomal instability, telomere dysfunction, dicentric, MSI, P53

## Abstract

*Background*: Microsatellite and chromosomal instability have been investigated in Hodgkin lymphoma (HL). *Materials and Methods*: We studied seven HL cell lines (five Nodular Sclerosis (NS) and two Mixed Cellularity (MC)) and patient peripheral blood lymphocytes (100 NS-HL and 23 MC-HL). Microsatellite instability (MSI) was assessed by PCR. Chromosomal instability and telomere dysfunction were investigated by FISH. DNA repair mechanisms were studied by transcriptomic and molecular approaches. *Results*: In the cell lines, we observed high MSI in L428 (4/5), KMH2, and HDLM2 (3/5), low MSI in L540, L591, and SUP-HD1, and none in L1236. NS-HL cell lines showed telomere shortening, associated with alterations of nuclear shape. Small cells were characterized by telomere loss and deletion, leading to chromosomal fusion, large nucleoplasmic bridges, and breakage/fusion/bridge (B/F/B) cycles, leading to chromosomal instability. The MC-HL cell lines showed substantial heterogeneity of telomere length. Intrachromosmal double strand breaks induced dicentric chromosome formation, high levels of micronucleus formation, and small nucleoplasmic bridges. B/F/B cycles induced complex chromosomal rearrangements. We observed a similar pattern in circulating lymphocytes of NS-HL and MC-HL patients. Transcriptome analysis confirmed the differences in the DNA repair pathways between the NS and MC cell lines. In addition, the NS-HL cell lines were radiosensitive and the MC-cell lines resistant to apoptosis after radiation exposure. *Conclusions*: In mononuclear NS-HL cells, loss of telomere integrity may present the first step in the ongoing process of chromosomal instability. Here, we identified, MSI as an additional mechanism for genomic instability in HL.

## 1. Introduction

Hodgkin lymphoma (HL) is a unique disease in which the neoplastic Hodgkin and Reed–Sternberg (HRS) cells constitute only 1–2% of the tumor tissue, the remaining tumor microenvironment being composed of various inflammatory cells [1]. Understanding the pathology of HL and developing rational therapies requires identification of the mechanisms underlying oncogenic evolution. Several aberrantly activated pathways have been identified in HRS cells [2]. Nevertheless, no recurrent chromosomal rearrangements have been found [3,4] and the functional status of *TP53* is still a matter of debate [5,6]. However, genomic instability has been described as a characteristic of HRS cells and a driving force of the aggressiveness of HL [7,8,9]. The mechanisms underlying such genomic instability are still unclear [8].

Two important models have been implicated in the genomic instability observed in cancer cells: microsatellite instability (MSI) and chromosomal instability, of which the latter inversely correlates with MSI in hereditary non-polyposis colorectal cancer [10] and determines the specific therapeutic strategy [11]. 

Only a single study has previously investigated the implication of MSI in HL, and concluded that MSI is not involved in the oncogenic process [12]. Nevertheless, chromosomal instability, i.e., multiple structural and numerical chromosomal rearrangements that are related to telomere dysfunction, has been consistently reported for HL patients and HL cell lines [13,14,15,16]. The transition from mononuclear to binuclear cells in HL has been associated with telomere loss [14]. The occurrence of complex chromosomal abnormalities and hyperploidy has been observed in HRS cells [14,17,18]. The mechanisms which drive such somatic chromosomal changes in HRS cells are yet to be elucidated. The precise role of telomere dysfunction, dicentric chromosome formation, and aneuploidy in generating chromosomal complexity and ongoing genomic instability are still unclear for HL. Molecular studies of HL face the problem of high heterogeneity in HL lymph nodes due to the unique and heterogeneous microenvironment of the HRS cells in HL [19]. In addition, cytogenetic analyses of primary Hodgkin tumors are hampered by the lack of in vitro growth of the tumor cells and the absence of suitable animal models. Thus, we used cell lines derived from malignant HRS cells and circulating lymphocytes of HL patients for these studies [20]. 

The aim of this study was to investigate mechanisms underlying genomic instability in HL through combined cytogenetic and molecular approaches. We demonstrate, for the first time, the involvement of MSI in HL cell lines. The loss of the protective function of telomeres in NS-HL cell lines induced chromosomal fusions (dicentric chromosome formation) and “breakage-fusion-bridges” (B/F/B) cycles, resulting in a series of chromosomal breaks and duplications, which can lead to chromosome imbalances, gene amplification, non-reciprocal translocation, and altered gene expression. In MC-HL cell lines, the high level of spontaneous double strand breaks (DSB) within and outside telomeres, detected using 53BP1 foci, induced the formation of dicentric chromosomes and the lagging of acentric chromosomes (micronuclei with only telomere sequences) and B/F/B cycles. Transcriptome analysis demonstrated the difference in DNA repair mechanisms between the NS-HL and MC-HL cell lines. Finally, a NS-HL cell line exhibited high radiation sensitivity compared to MC-HL cell line. In addition, we validated our findings in a large cohort of NS-HL and MC-HL patients.

## 2. Results

### 2.1. Genomic Instability in HL Cell Lines via Microsatellite Instability and P53 Status

Table 1 shows the results obtained after the screening for MSI using five quasimonomorphic mononucleotide repeats. These results demonstrate the absence of MSI in L1236 (0/5), low MSI (MSI-L) (1/5) in L591, SUP-HD1 and L540, and high MSI (MSI-H) (more than 3/5) in HDLM2, KMH2, and L428. In HL cell lines (95.5% for L428, 95.3% for KMH2, and 92.3% for HDLM2), we found a correlation between MSI and the co-expression of CD30+/CD15+, one of the clinical hallmarks of HL. (Figure S1). 

Analysis of the functional status of TP53 (cf. Materials and Methods section) showed the presence of 100% red colonies generated from three of the cell lines (L428, L1236, and HDLM2), indicating the nonfunctional status of *TP53* and the clonal homogeneity of these cell lines (Figure S2A). Sequencing of p53 cDNA confirmed the presence of the mutations in L428 (exon4), L1236 (exon 10–11), and HDLM2 (exon 8–11), in agreement with a previously published study [21]. FISH analysis for the *TP53* gene also revealed a deletion of one allele of *TP53* in HDLM2 and a high copy numbers in the L428 cell line were associated with *TP53* breakpoint rearrangement (Figure S2B). 

### 2.2. Genomic Instability via Chromosomal Instability in HL Cell Lines

#### 2.2.1. Telomere Dysfunction in HL Cell Lines

**Figure 1:**
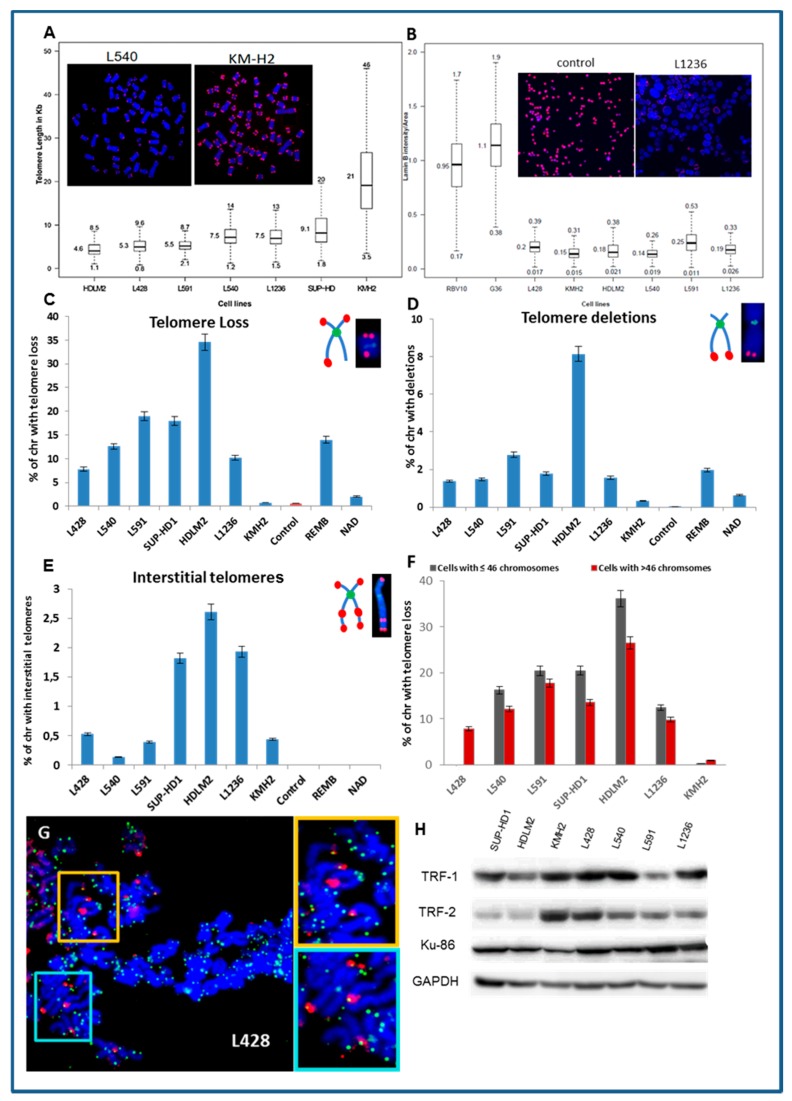
Telomere dysfunction in Hodgkin lymphoma (HL) cell lines (**A**) Quantification of telomere length by teloquant-Q-FISH with specific PNA probes. In the box plots of telomere length, the midline reflects the median, the box length the interquartile range (interquartile range, 25th to 75th percentile), and the whiskers the 5th and 95th percentiles. The Min and Max values are presented. The images are of an L540 metaphase with short telomeres and one of KMH2 with long telomeres (63× magnification). (**B**) Box plots of the fluorescence intensity of Lamin B1 protein in HL cell lines and control lymphoblastoid cell lines RV10 and G36. The midline reflects the mean intensity. An image of the fluorescent signal of lamin B1 in nuclei of L1236 HL cells and control cells is presented (10× magnification). (**C**) Telomere loss, (**D**) telomere deletion, and (**E**) interstitial telomeres were scored in HL cell lines, in two lymphoblastic cell lines (REMB and NAD), and circulating lymphocytes from healthy donors, serving as a control. (**F**) Telomere loss in small cells (≤46 chromosomes) compared to that of large cells (more than 46 chromosomes). (**G**) Representative images obtained following IF-FISH. TIFs (yellow) represent co-localization of γH2AX (red) foci and telomere (green) signals in the L428 cell line (63× magnification). (**H**) Western blot analysis of proteins involved in telomere maintenance (TRF1, TRF2, and Ku86) demonstrate diminished levels of TRF2 in HDLM2 and SUP-HD cells, TRF1 in L591, and Ku86 in KMH2. GAPDH was used as a loading control.

**Figure 2:**
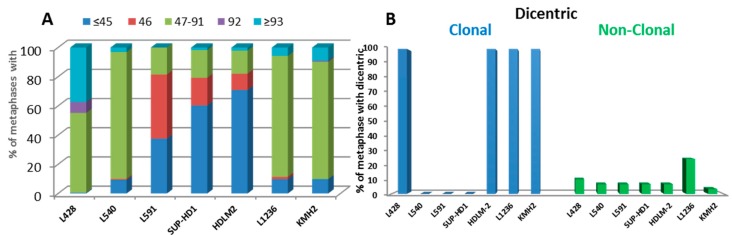
Numerical and structural aberrations detected using telomere (red) and centromere (green) staining, followed by M-FISH in HL cell lines. (**A**) Numerical chromosomal aberrations were determined after scoring 200 metaphases (**B**) The presence of clonal and sporadic dicentric chromosomes in HL cell lines.

We observed telomere shortening (less than 6 kb) in three cell lines (HDLM2, L428, and L591) (Figure 1A). Only KMH2 cells exhibited a high mean telomere length (21 kb). Telomere length was significantly different between small and large cells (Figure S3) and associated with the irregularity of the nuclei (Figure S4) and very low lamin B1 expression, implicated in nuclear shape alterations and telomere dysfunction (Figure 2B). Large cells exhibited telomere shortening, a high frequency of irregular nuclei, and low level lamin B1 expression. 

The use of Q-FISH permits the analysis of telomere aberrations: telomere loss, telomere deletions, and interstitial telomeres in metaphases. We observed a high rate of telomere loss and deletion in the HL cell lines and a lymphoblastoid cell line (REMB) with short telomeres (Figure 1C). Nevertheless, the presence of interstitial telomeres was exclusively detected in the HL cell lines (Figure 1E). Small cells (≤ 46 chromosomes) showed a higher frequency of telomere loss and deletion than large cells (Figure 2F). These telomere dysfunctions were associated with the presence of spontaneous γH2AX foci co-localized with telomeres (Figure 1G). TRF2, TRF1, and Ku86 are involved in telomere maintenance as well as in DNA DSB repair. To assess the relationship between telomere dysfunction and expression levels of these proteins, western blot analyses were carried out. The HL cell lines exhibited various levels of these proteins (Figure 1H). TRF2 expression was low in the HDLM2 and SUP-HD cell lines and TRF1 in L591. Only, KMH2 exhibited low levels of Ku86 (Figure 1H).

#### 2.2.2. Aneuploidy, Dicentric Chromosomes, and Micronucleus Formation Lead to Chromosomal Instability in HL Cell Lines

All HL cell lines demonstrated numerical chromosomal aberrations (Figure 2A). In the L591 cell line, only 40% of the diploid cells had no loss or gain of chromosomes. Thus, aneuploidy represents the main common cytogenetic characteristic of HL cell lines (Figure S5).

The second common characteristic was the presence of clonal and sporadic dicentric chromosomes (Figure 2B). Only the HDLM2, L1236, KHM2, and L428 cell lines exhibited clonal dicentric chromosomes. These dicentric chromosomes were not associated with the presence of fusion acentric chromosomes, except in KMH2 metaphases. Nevertheless, terminal acentric chromosomes were identified and correlated with the presence of sporadic dicentric chromosomes (Figure S6). 

**Figure 3:**
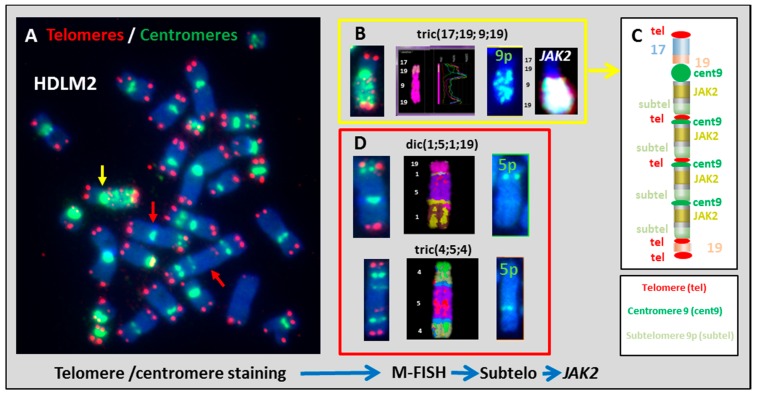
Dicentric chromosome formation in HDLM2. (**A**) Telomere and centromere staining show the presence of several clonal dicentric chromosomes (100× magnification). (**B**) tric(17;19;9;19) contained several centromeric, telomeric, and subtelomeric sequences of 9p. *JAK2-*specific *FISH* revealed amplification of this gene. (**C**) This approach makes it possible to schematize this chromosome resulting from B/F/B cycles. (**D**) Two other clonal dicentric chromosomes were analyzed using the same approach: dic(1;5;1;19) and tric(4;5;4) and the presence of subtelomeric sequences of 5p was also confirmed in these two dicentric configurations with complex chromosomal rearrangements.

We sought to better understand the mechanisms of dicentric chromosome formation in HL cell lines by first assessing the involvement of telomere dysfunction in this phenomenon. In the HDLM2 HL cell line, chromosomes 5 and 9 were involved in clonal dicentric chromosome formation. Sequential analyses, by telomere and centromere staining followed by sub-telomere hybridization, identified the presence of sub-telomere sequences at the breakpoints of dicentric or tricentric chromosomes in HDLM2 (Figure 3). In addition, interstitial telomeres were detected in the same chromosomes. We detected centromeric, interstitial telomere, and subtelomere sequences of chromosome 9p in tric(17;9;19). *JAK2*-specific FISH revealed the presence of several gene copies (Figure 3B). This analysis allowed us to classify, for the first time, this derivative chromosome. Moreover, this analysis also demonstrated the involvement of telomere dysfunction in complex chromosomal rearrangement and gene amplification as a result of B/F/B cycles (Figure 3C). We observed the same mechanism in the formation of dic(1;5;1;19) and tric(4;5;4) (Figure 3D). The presence of subtelomeric sequences of 5p in the breakpoint of dicentric chromosomes demonstrate that these aberrations were related to telomere dysfunction and chromosome fusions, which induced B/F/B cycles and the formation of complex chromosomal aberrations.

**Figure 4:**
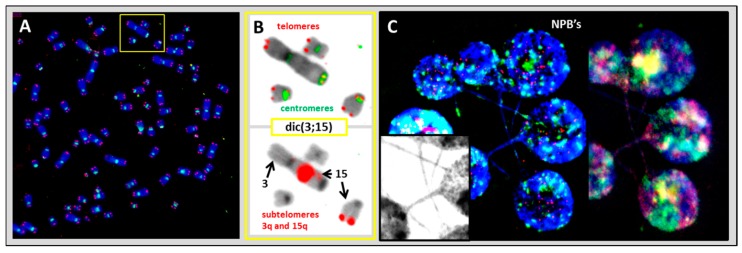
Clonal dicentric chromosome in the L428 cell line. (**A**) Telomere (red) and centromere (green) staining followed by (**B**) subtelomere staining of 3q (red) and 15q (red) demonstrated the presence of subtelomeric sequences in the breakpoint of this dicentric chromosome (100× magnification). (**C**) Large nucleoplasmic bridge (NPB’s) with centromeric sequences were connected to the daughter cells even after four days of culture in the presence of cytochalasin B. M-FISH demonstrates the presence of chromosomes 3 and 15 in NPB’s (63× magnification).

We analyzed the dicentric chromosomes in the L428 cell line using the same strategy. We identified dic(3;15) and found the subtelomeric sequences of 15q to be in the breakpoint of the dicentric chromosome (Figure 4A,B), showing that this dicentric chromosome arises from telomere end fusions caused by telomere dysfunction, with the loss of telomeric sequences in chromosomes 3q and 15q. In addition, large nucleoplasmic bridges (NPB’s) containing centromere sequences were connected to the daughter cells within the micronuclei, confirming that the observed dicentric chromosomes in L428 resulted from telomere and telomere end fusion (Figure 4C). 

**Figure 5:**
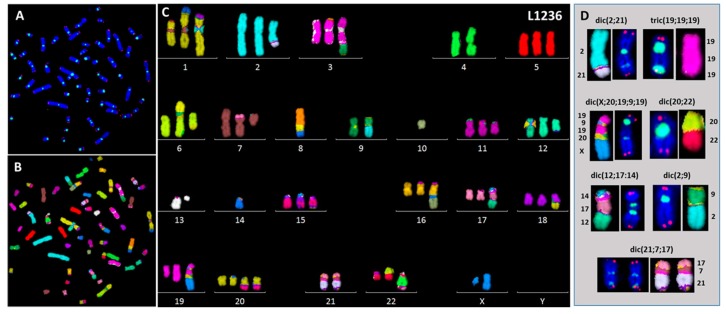
Breakpoints in clonal dicentric chromosomes in L1236 cells by sequential analysis using (**A**) telomere and centromere staining followed by (**B**) M-FISH staining and (**C**) classification of chromosomes. (**D**) Pericentromeric breakpoints detected in clonal dicentric chromosomes (63× magnification).

We used the same approach to study the MC-HL cell lines, and lack of sub-telomeric sequences in dicentric chromosome breakpoints was observed also in L1236 and KHM2 cells (Figure S7). Nevertheless, sequential analysis by telomere and centromere staining (Figure 5A) and M-FISH (Figure 5B) revealed that the breakpoints of dicentric chromosomes in L1236 cells were localized in centromeric or pericentromeric regions (Figure 5C,D). Interestingly, telomere shortening in L1236 cell line may contribute to the formation of terminal translocation with telomeric breakpoints, i.e., rearrangement in chromosome 16, 17, 18 and 20 (Figure 5C). 

**Figure 6:**
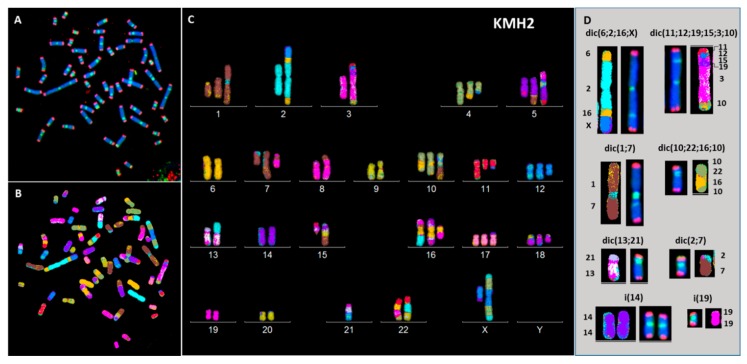
Breakpoints in clonal dicentric chromosomes in KMH2 cells. (**A**) telomere and centromere staining followed by (**B**) M-FISH and (**C**) chromosome classification revealed (**D**) pericentromeric breakpoints in dicentric chromosomes (63× magnification).

**Figure 7:**
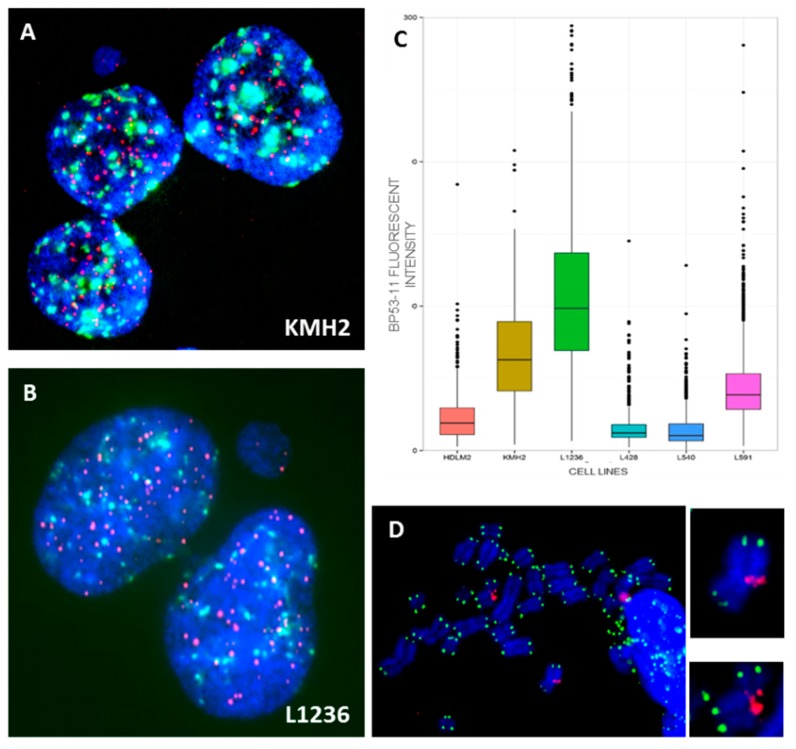
(**A**) Small nucleoplasmic bridge without staining connected the daughter cells after four days of culture in the presence of cytochalasin B in KMH2 and (**B**) in L1236 cells. (**C**) Total intensity of spontaneous 53BP1 foci in HL cell lines. (**D**) Representative images obtained following IF-FISH in KMH2 cells showing the presence of γH2AX (red) foci in intra-chromosomal region. Telomeres were stained with green signals (63× magnification).

Clonal dicentric chromosomes in KMH2 cells were characterized by a specific configuration with the pericentrometric breakpoints and the two centromeres in close proximity (Figure 6). In addition, the presence of small NPB’s, associated with micronuclei containing telomeric sequences only, originating from the acentric chromosome (Figures S4–S8), were observed in these cell lines (Figure 7A,B). Of note, we observed high expression of 53BP1 protein in the L1236 and KMH2 cell lines (Figure 7C), in which we found intra-chromosomal γH2AX foci (Figure 7D).

#### 2.2.3. Structural Chromosome Aberrations

The clonal structural chromosomal aberrations identified in each cell line are summarized in Table S1.

Numerical chromosomal aberrations were present in all HL cell lines, with a higher frequency of gains than losses. We observed the loss of chromosome Y in four cell lines derived from men. Chromosome 13 was lost from five cell lines and chromosomes 15 and 8 from two. There were gains for chromosomes 19 (6/7), 9(6/7), 5 (5/7), 11 (5/7), 18 (5/7), and 17 (4/7) (Figure S9).

**Figure 8:**
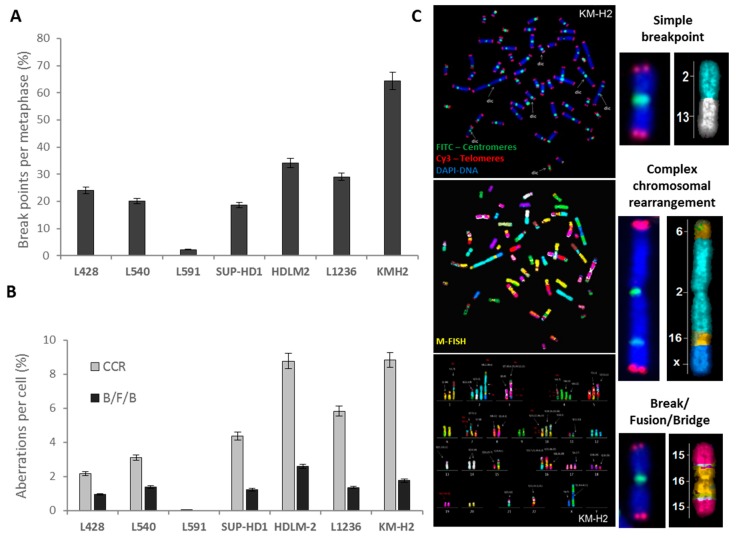
Chromosomal instability detected by M-FISH. (**A**) Frequency of total breakpoints in HL cell lines. (**B**) Frequency of complex chromosomal aberrations (CCRs) and break fusion Bridges (B/F/Bs). (**C**) Metaphase hybridized with telomere and centromere PNA probes and subsequently M-FISH probes, permitting the precise classification of the chromosomes and the establishment of the karyotype. This metaphase contained simple chromosomal aberrations, CCRs, and B/F/Bs. (63× magnification).

The total frequency of breakpoints resulting from all aberrations detected by sequential analysis, using telomere and centromere staining followed by M-FISH, are presented in Figure 8A. All HL cell lines, with the exception of L591, showed complex chromosomal rearrangements (CCRs) and B/F/B aberrations (Figure 8B) associated with a high frequency of total breakpoints. No shared structural aberrations were found in the HL cell lines. Nevertheless, we identified der(2)t(2;8), without the fusion gene of *MYC*, in all the NS -HL cell lines (Figure S10A) and its amplification was observed primarily in L428 and HDLM2 cells. In addition, L428 and L591 cells contained t(9;14). Interestingly, we observed an elevated number of copies of *JAK2* in 6/7 cell lines. HDLM2 exhibited a particularly high copy number for *JAK2* (eight copies) (Figure S10B), including three copies of chromosome 9 alone, induced by B/F/B cycles.

There was no correlation between chromosomal instability and *TP53* mutation nor MSI instability and telomere shortening in the HL cell lines. Nevertheless, the NS HL cell lines with high MSI showed a high frequency of CCRs and B/F/B aberrations. In addition, KMH2, a MC HL cell line, showed a high frequency of CCRs relative to L1236 cells. 

## 3. Validation of Telomere Dysfunction Findings in Peripheral Blood Lymphocytes of HL Patients

**Figure 9:**
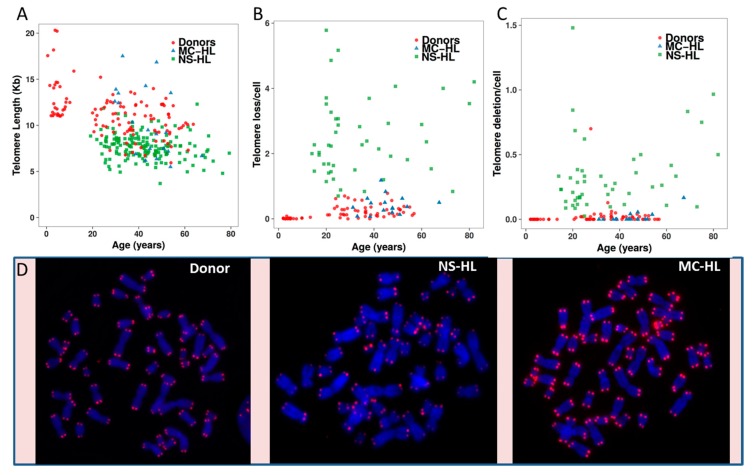
Telomere dysfunction in NS-HL (square green) and MC-HL patients (triangle blue) and healthy donors (circle red). (**A**) Telomere length (kb). (**B**) Telomere loss. (**C**) telomere deletions. (**D**) Representative image for a metaphase of a healthy donor, NS-HL patient, and MC-HL patient (63× magnification).

We examined telomere dysfunction in circulating lymphocytes from 100 NS-HL and 23 MC-HL patients, as previously published [15,22], to validate our observations in HL cell lines that telomere dysfunction (length and aberrations) may play a major role in NS-HL. We found a significant difference between: (1) telomere length, telomere loss, and telomere deletions in HL patients and healthy donors (*p* < 10^−16^; *p* < 10^−16^; *p* < 10^−9^, respectively) and (2) telomere length, telomere loss, and telomere deletions in NS-HL and MC-HL patients (*p* < 10^−2^; *p* < 10^−16^ and *p* < 10^−16^, respectively) (Figure 9). Interestingly, telomere length heterogeneity, one of the characteristic of ALT mechanisms, was more pronounced in MC-HL patients than that observed in NS-HL. A subset of MC-HL patients had long telomeres relative to those of healthy donors. There was no significant difference between telomere length and telomere deletions in MC-HL patients relative to healthy donors. The only significant difference observed was the frequency of telomere loss between MC-HL patients and healthy donors (*p* < 10^−5^). These characteristics reflect our observations in the HL cell lines and the long telomeres observed in KMH2 cells.

## 4. Transcriptome Analysis

**Figure 10:**
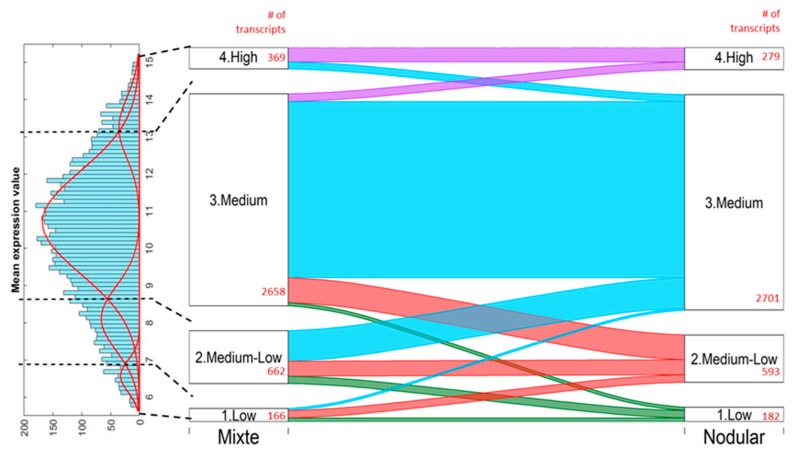
Differentially expressed transcripts between NS and MC cell lines. The transcripts were divided into four groups based on expression level (Low, Medium-Low, Medium, and High) by Gaussian Mixture Modelling. The histogram of mean expression values of the cell lines (on the left) was decomposed to four components based on BIC criteria, resulting in thresholds dividing the groups (black dashed lines on the histogram by Gaussian Mixture Modelling [23]. The number of transcripts were sought for each group for the MC and NS cell lines, separately (red numbers). The Sankey plot on the right shows the relation between the number of transcripts, by group, in two cell lines: the wider the ribbon, the more transcripts fall into the proper category.

We restricted the transcriptome analysis in this study to the comparison of NS HL with MC HL cell lines. We found 3,755 transcripts and 2,415 unique genes to be differentially expressed between the NS-HL (L428, HDLM2, L591 and L540) and MC-HL (L1236 and KHM2) cell lines (Figure 10). The affected pathways, including metabolic, cellular, single-organism, and regulation processes and biological regulation may be found in Figure S11.

Functional analysis revealed a significant difference in DNA repair pathways as defined by KEGG between NS-HL and MC-HL cell lines. There was a significant difference in the expression of 140 transcripts, with 78 genes corresponding to DNA repair pathways (*p* = 0.005) (Table S2).

**Figure 11:**
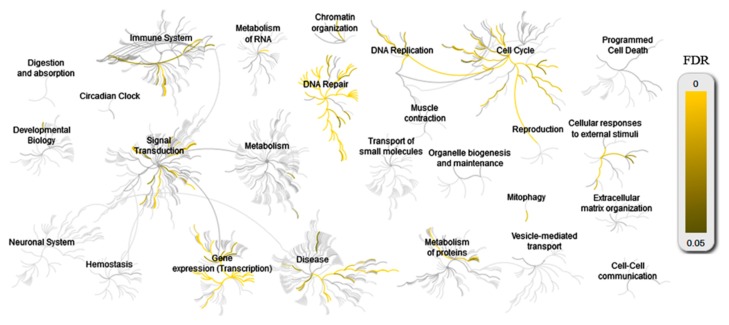
Over-representation analysis of Reactome pathways. The plots are separately made for differentially expressed genes between NS and MC-HL cell lines for DNA repair ontology.

DNA repair ontology and the affected pathways are part of a Reactome pathway (Figure 11). DNA repair transcripts have been identified in DNA repair, cell cycle, disease, the immune system, and signal transduction (Figure 11A). A large significant difference was observed for PML expression between NS-HL and MC-HL cell lines. The overexpression of PML in MC-HL was confirmed by western blotting and immunofluorescence [24], as well as the overexpression of *MRE11* by immunofluorescence.

## 5. Altered Double-Strand Break Response in HL Cell Lines

**Figure 12:**
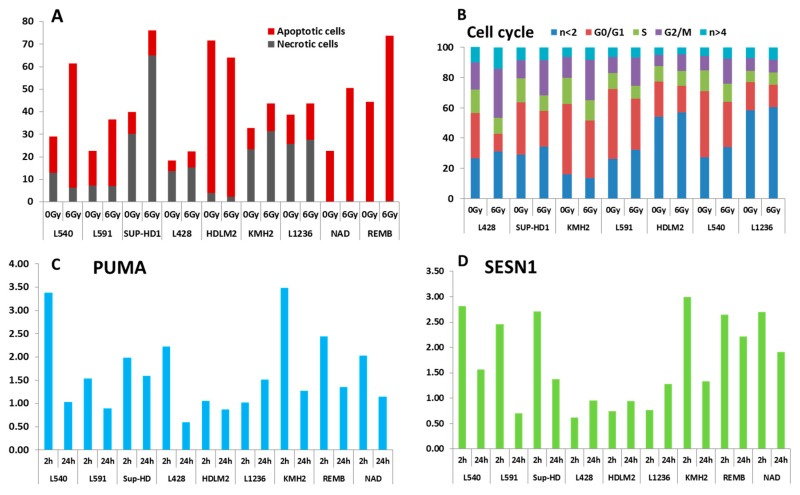
Radiation sensitivity of HL cell lines. (**A**) Apoptotic and necrotic cells after 24 h of exposure to 6 Gy. Lymphoblastoid B cell lines (REMB and NAD) where used as a control. (**B**) Analysis of the cell cycle after 24 h of exposure to 6Gy. (**C**) PUMA and (**D**) Sesterin 1 (SESN1) gene expressions were analyzed at 2 and 24 h after 2Gy irradiation by quantitative RT-PCR. Values are normalized to HPRT expression levels. The gene expression ratio of irradiated/mock-treated cells is presented. The mean values of triplicate experiments, each with three RT-PCR reactions, are shown.

The presence of two different mechanisms underlying genomic and chromosomal instability in HL cells suggests that the existence of a different pathway in DNA repair may be an important biological event that influences treatment resistance in HL. Therefore, we explored the radiation sensitivity of the HL cell lines. The HL cell lines and two lymphoblastoid B cell lines (NAD and REMB) were exposed to ionizing radiation and two approaches were used to assess radiation sensitivity: (a) apoptosis, including cell cycle arrest after 6Gy of irradiation by FACs analysis, and (b) mRNA expression of PUMA, P21, ZMAT3, Sesterin1 (SESN1), and CCNG1 at two and 24 h after 2Gy of irradiation [25]. These genes are involved in DNA repair: PUMA is a p53 upregulated modulator of apoptosis; P21 a regulator of cell cycle progression at G1; ZMAT3 encodes a protein containing three zinc-finger domains and over expression of this gene inhibits tumor cell growth; SESN1 plays a role in the cellular response to DNA damage and oxidative stress-induced over expression inhibits tumor cell growth, and the expression of this gene is a potential marker for exposure to radiation; and CCNG1 encodes a human homologue of the rat G-type cyclin. Lymphoblastoid B cells displayed a normal response to ionizing radiation, characterized by nearly complete mitotic arrest, preceding massive apoptosis (Figure 12A), and high mRNA levels of all the genes investigated (Figure 10C,D). For the HL cell lines, we identified three groups. The first group, characterized by a G2 arrest phenotype, was sensitive to apoptosis following irradiation (L540 and L591). The second group (L428, SUP-HD, and KMH2) was characterized by the resistance to induce apoptosis after irradiation, but G2 arrest was observed in these cell lines (Figure 12B). The third group (L1236, and HDLM2) was characterized by a high spontaneous sub-G1 fraction and resistance to induced apoptosis after irradiation. For HDLM2, we observed a high level of apoptotic cells prior to irradiation and the difference after irradiation was not evident. mRNA expression data (Figure 12C,D) also demonstrated a significant difference between the response of these three groups of cell lines relative to lymphoblastoid cell lines. The expression of P21 (Figure S12) and SESN1 (Figure 10D) were associated with the TP53 mutation. Nevertheless, the L428 cell line showed a significant response for PUMA expression (Figure 12C).

## 6. Discussion

The mechanisms underlying genomic instability and the primary transforming events of HL are still obscure [8,9]. In this study, we assessed the involvement of two important mechanisms of genomic instability: MSI and chromosomal instability.

We report, for the first time, that MSI is involved in HL. We confirmed the results obtained for L1236 concerning the absence of MSI [12] and demonstrated a high frequency of MSI in L428, KMH2, and HDLM2 cells. We propose MSI as an additional mechanism of genomic instability in some HL cell lines. It will be informative to investigate MSI in HL lymph nodes to establish not only the correlation between MSI and clinical outcomes of patients treated with standard therapy, but also to develop new therapies and personalized treatment. MSI-cancers may be excellent candidates for immune checkpoint inhibitors [11,26,27].

The *TP53* status of HL has been the subject of debate and remains controversial [21]. The lack of proven *TP53* mutations in lymph nodes contrasts with the levels of its expression which are often high [6]. The disruption of this process can promote tumor progression and resistance to treatment [28]. In this study, functional yeast assays revealed the non-functionality of *TP53* in three cell lines and DNA sequencing revealed the same mutations previously described in L1236, HDLM2, and L428 [21]. This re-evaluation of the *TP53* status in HL using a sensitive technique reinforces the concept that *TP53* mutation may be involved in the pathology of some cases of HL [29] and perhaps in the genomic instability observed in this disease, as well as the occurrence of late complications, such as secondary cancer following HL treatment [30] or HL as a secondary event [6]. It may be informative to investigate the *TP53* status in lymph nodes derived from relapsing or refractory HL patients. The correlation between *TP53* status and high-grade progression in B-cell lymphoma has been previously established [31,32], as well as the resistance to treatment. Of note, the two NS-HL cell lines (L428 and HDLM2) with *TP53* mutation were characterized by high MSI. For MC-HL, we did not detect MSI in the L1236 cell line, which carries mutated *TP53*, and conversely, we detected a high frequency of MSI in the KMH2 cell line, without a *TP53* mutation.

Chromosomal instability, a second mechanism of genomic instability, has been defined as an accumulation of point mutations, translocations, chromosomal gains and losses, telomere shortening, and defects in the nuclear architecture, which may cause genome instability [33].

Here, we demonstrate the involvement of telomere dysfunction in chromosomal instability in HL. We first confirmed previous published data using seven HL cell lines and a large cohort of HL patients [14,15] and validated telomere shortening associated with the high heterogeneity observed in HL patients relative to healthy donors. This was similarly observed in HL cell lines, which were characterized by a high level of telomere length heterogeneity and progressive telomere loss between mononuclear and binuclear cells [14], associated with nuclear irregularity and a low level of lamin B1 [34] which could be related to this telomere dysfunction.

Second, we demonstrate, for the first time, a significant difference between telomere length in NS-HL and MC-HL patients and cell lines. MC-HL patients showed high heterogeneity and a subset of patients showed long telomeres, as well as the KMH2 cell line. All NS-HL patients and cell lines presented telomere shortening associated with a high frequency of telomere aberrations (loss of one or two telomeres). Mononuclear cells presented longer telomeres than RS cells, but a higher frequency of telomere loss. We speculated that the loss of telomere integrity of mononuclear cells in NS-HL cell lines may represent the first step in the ongoing process of chromosomal instability. Such telomere dysfunction induced the formation of dicentric chromosomes, which have been observed in oncogenic processes, particularly hematological disorders [35].

In the HDLM2 and L428 cell lines, we identified the subtelomeric sequences at the breakpoint of clonal dicentric chromosomes, in addition to the presence of interstitial telomere sequences. These dicentric chromosomes developed into large chromatin bridges connecting the daughter cells. The chromatin bridges did not appear to give rise to micronuclei [36], but contained the centromere and telomere sequences [37]. These processes induced chromosomal instability via B/F/B cycles, leading to complex chromosomal rearrangements and the amplification of genes such as *Jak2* in the HDLM2 cell line. The findings reported here suggest that chromosomal instability in NS-HL cell lines can arise as a consequence of telomere dysfunction in the early stages of HL tumorigenesis. HRS cells, the end of the proliferative stage, were characterized by telomere shortening, the accumulation of complex chromosomal rearrangements, and fewer telomere aberrations, resulting in a stable stage. Of note TP53 mutation, detected in L428 and HDLM2 cell lines, play a role in telomere shortening as well as the accumulation of chromosomal rearrangements and chromosomal instability [31,38,39].

Nevertheless, the mechanisms of dicentric formation in MC-HL cell lines are not related exclusively to telomere dysfunction. The lack of sub-telomere sequences at the dicentric breakpoints which were usually localized to the pericentromeric region, discards the possibility that telomere dysfunction exclusively induced the formation of dicentric chromosomes in the MC HL cell lines. We propose that spontaneous DNA DSBs detected by IF-FISH and associated with a higher frequency of 53BP1 foci, were produced in pericentromeric regions due to cellular metabolism or a genetic program, and induced the formation of dicentric chromosomes with specific configurations. Nucleoplasmic bridges without signals associated with the presence of micronuclei with only telomere sequences (terminal deletions) may lend support to this hypothesis. These dicentric chromosomes contributed to the subsequent accumulation of CCRs and alterations related to B/F/B cycles. This profile was found in KMH2 cells. However, in L1236, the *TP53* mutations plays a major role in telomere dysfunction [39] and the involvement of telomeres was not excluded in the cell line. Interstitial telomeres were observed in this cell line.

Dicentric chromosomes that have been observed in oncogenic processes, in particular in hematological disorders [35,40,41,42,43], are considered to be unstable chromosomal aberrations and disappear with successive cell divisions, with the natural occurrence of this aberration being very low [44]. In rare cases, dicentric chromosomes can be stably maintained by inactivation of one of the centromeres [45,46,47,48,49], telomerase or break-induced replication [48,50,51] or specific dicentric chromosome configurations related to telomeric or centromeric breakpoints [52,53]. Here, we demonstrate that the presence of clonal dicentric chromosomes in L428 and HDLM2 cell lines is related to chromosome fusion and telomere breakpoints. Nevertheless, dicentric chromosomes in L1236 and KMH2 cells were associated with peri-centromeric breakpoints. We detected higher telomerase activity in L428 and HDLM2 than L1236 and KMH2 cells [24], suggesting that dicentric chromosome formation was associated with telomere dysfunction and high telomerase activity. 

The response to ionizing radiation is associated with the *TP53* status and MSI and thus the type of HL cell line. We demonstrate, for the first time, the radiation sensitivity of NS-HL cell lines and confirm the relevance of radiation therapy in HL treatment [54]. The L540, L591, and SUP-HD cell lines were more radiosensitive than the L428 and HDLM2 cell lines. The response of the HDLM2 cell line to radiation may be related to the age of the patient (74 years) from which this cell line was derived and the generally poor clinical outcome of older HL patients [55]. Nevertheless, the L428 cell line showed G2 arrest, despite the TP53 mutation. In MC-HL cell lines, both cell types were resistant to apoptosis, but the KMH2 cell line, with greater MSI, showed G2-arrest. We observed no significant effects of radiation for L1236. Future studies will require delineation of the in vivo radiation sensitivity of NS-HL versus MC-HL patients, including the role of TP53 and MSI in the response to treatment [6].

Transcriptome analysis confirmed this difference between the NS and MC HL cell lines with respect to DNA repair processes, telomere maintenance, and cell cycle and apoptotic process.

## 7. Materials and Methods

### 7.1. Cell Lines Used, Patients and Culture Conditions

The human HL-derived cell lines L428 [56], L591 [56], SUP-HD1 [57], L540 [56], HDLM2 [58], L1236 [59], and KMH2 [60], were obtained from the German Collection of Microorganisms (Braunschweig, Germany). The origin and histological characteristics are shown in Table 2.

Peripheral blood lymphocytes were obtained from 100 patients with NS HL and 100 healthy donors following informed consent (Table 3). The collection of blood samples from patients and donors was approved by the Ethics Committee of Gustave Roussy Cancer Campus University Paris Saclay (approved number 97-06). All patients were treated by combined modalities (chemotherapy and radiation therapy). Cytogenetic preparations were carried out from NS HL patients and healthy donors.

The HL cell lines and in vitro Epstein-Barr virus (EBV) infected cell lines NAD, REMB, G36, and RV10 were cultured at 37 °C in an atmosphere containing 5% CO_2_ in RPMI 1640 medium (Gibco, Grand Islands, NY, USA) supplemented with 10% fetal bovine serum (Eurobio, Courtaboeuf, France) and 1% antibiotic-antimycotic (Gibco).

### 7.2. Cytogenetic Slides (Preparation of Metaphase Spreads)

Cells were exposed to colcemid (0.1 µg/mL) (Gibco KaryoMAX,) for 2 h at 37 °C, 5% CO_2_, in a humidified atmosphere to block them in metaphase. After harvesting the cells and centrifugation for 7 min at 1400 RPM at room temperature, the supernatant was removed and the cells were re-suspended in a solution of warm (37 °C) 0.075 M potassium chloride (KCl) (Merck, Kenilworth, NJ, USA) and incubated for 20 min in a 37 °C water bath (hypotonic shock). For pre-fixation of the cells, approximately five drops of fixative (3:1 ethanol/acetic acid) was added to each tube under permanent agitation and the tubes centrifuged for 7 min at 1400 RPM at room temperature. The supernatant was removed and the cells suspended in fixative solution and centrifuged using the same parameters. After two additional rounds of these fixative steps, the cells were stored in fixative solution at 4 °C overnight and metaphases were spread on cold, wet slides the next day. Slides spread with metaphases were dried overnight at room temperature and stored at −20 °C until further use.

### 7.3. CD30 and CD15 Detection and Cell Cycle Analysis by Flow Cytometry

Immuno-phenotyping of Hodgkin lymphoma cell lines was performed on a LSRII flow cytometer (BD) using FITC- and APC-conjugated mouse anti-human specific monoclonal antibodies for CD30 (Ber-H83) and CD15 (HI98), respectively. Both antibodies were obtained from BD Biosciences (BD Biosciences, Le Pont de Claix, France). Staining for CD15 and CD30 was performed according to the manufacturer’s instructions. After the addition of 1 μg/mL Hoechst (BD PharMingen, Le Pont de Claix, France) to discriminate dead cells, the cells were analyzed by FACS as previously described.

Cell-cycle fractions were determined by Hoechst nuclear staining. Briefly, cells were harvested, washed in PBS, and incubated in Hoechst solution for 30 min at room temperature. Data were collected using a FACSCalibur flow cytometer (BD Biosciences) and analyzed using FlowJo Version 7.5.5 (FlowJo LLC, Ashland, OR, USA). The results represent the mean value of three independent experiments.

### 7.4. TP53 Functional Assay for Screening of the Cell Lines

To study the functionality of the TP53 gene, a functional assay was performed in accordance with the method described by Flaman et al. [61]. The assay tests the entire DNA-binding domain (aa 102–292), as the p53 expression vector is linearized at codons 67 and 346. Briefly, TP53 mRNA was reverse transcribed, amplified by PCR, and co-transfected into yeast with a linearized expression vector carrying the 5’ and 3’ ends of the p53 open reading frame. Gap repair of the plasmid with the PCR product results in the constitutive expression of human TP53 protein. Yeast cells that repaired the plasmid were selected on a medium lacking leucine. The medium contained sufficient adenine for growth of Ade^+^ cells, leading to the generation of white colonies. Thus, colonies containing wild-type TP53 were white (Ade2^+^) and those containing the TP53 mutation red. After identification of non-functional TP53 samples, red colonies were cloned and sequenced by the Sanger method to characterize the TP53 mutation. Finally, we classified the mutations using the IARC p53 database according to their impact on the protein.

### 7.5. Evaluation of Microsatellite Instability (MSI) in HL Cell Lines

The Revised Bethesda Guidelines were used to determine the MSI status. Analysis of MSI was performed by five-plex polymerase chain reaction (PCR) using a panel of five quasimonomorphic mononucleotide repeat markers (BAT26, BAT25, NR21, NR22, and NR24) that map to intron 15, intron 16, the 5′ untranslated region (UTR), 3′ UTR, and 3′ UTR of MSH2, c-kit, SLC7A8, transmembrane protein precursor B5, and ZNF2 genes. A single 5-plex polymerase chain reaction (PCR) allowed co-amplification of all five markers, which were subsequently analyzed using a genetic analyzer (ABI PRISM 310; Applied Biosystems, Courtaboeuf, France) and computerized fragment analysis for each of the five primer pairs used in the PCR.

The denatured PCR products were separated by capillary electrophoresis using the genetic analyzer and were further analyzed using commercially available software (GeneScan, Applied Biosystems, Foster City, CA, USA). HL cell lines with instability of three markers were defined as MSI-H, those with < 3 as MSI-L, or MSS if instability was not identified [62].

### 7.6. Immunofluorescence and Immunofluorescent-FISH (IF-FISH)

Cells were cytospun onto poly-l-lysine-coated glass slides at 700 rpm for 4 min, fixed with 10% formalin for 10 min, and treated with 0.25% Triton X-100 solution for 10 min. After blocking with 5% bovine serum albumin (Sigma Aldrich, Saint Quentin Fallavier, France), the cells were incubated overnight in 4 °C with primary antibody. After washing in PBS, cells were treated with Cyanine 3 anti-mouse IgG (Invitrogen, Carlsbad, CA, USA) or FITC anti rabbit (Sigma-Aldrich, Saint Quentin Fallavier, France) secondary antibody at 37 °C for 45 min. cells were mounted with p-phenylene diamine after counterstaining with 4,6-diamidino-2-phenylindole (Sigma-Aldrich). As a negative control, staining was carried out in the absence of primary antibody.

IF-FISH was performed using a protocol similar to one described previously [63,64]. Cells were centrifuged at 1000× *g* after 5 h of colchicine (0.09 mg/mL) treatment at 37 °C in a humidified atmosphere of 5% CO_2_. The pellet was washed in 1× PBS at 37 °C and re-centrifuged. The cells were subjected to hypertonic shock by resuspension in 34 mM citrate at 37 °C to obtain a suspension of cells at a concentration of 60,000 cells/mL and incubated for 1 h at 37 °C. The suspension (200 μL) was subsequently applied to polylysine slides by cytospin. Following fixation (paraformaldehyde (PFA) 3%, sucrose 2%), cells were immunostained as described. Prior to telomere hybridization with the PNA probe (CCCTAA)3-FITC, cells were successively fixed (PFA 4%, 2 min), washed in PBS, and dehydrated (50/70/100 ethanol).

### 7.7. Telomere Quantification

Telomere quantification was performed using the Q-FISH technique with a Cy-3-labelled PNA probe specific for (TTAGGG) (Eurogenetec, Liege, Belgium). Two approaches were developed. The first approach consisted of the quantification of telomere length in interphase cells, permitting the investigation of intercellular variation in a large number of scored cells. Quantitative image acquisition and analysis were performed using Metacyte software (Metasystem, version 3.9.1, Altlussheim, Germany). The mean fluorescence intensity (FI) of telomeres was automatically quantified in 10,000 nuclei on each slide. Settings for exposure and gain remained constant between captures. The experiment was performed in triplicate. The second approach consisted of quantifying telomere length in metaphases using automated acquisition module Autocapt software (MetaSystems, version 3.9.1) and a ZEISS Plan-Apochromat 63×/1.40 oil and CoolCube 1 Digital High Resolution CCD Camera with constant settings for exposure and gain. The mean telomere length was measured and telomere loss and telomere doublet scoring were performed. This approach allows the study of intra-cellular variation of telomere length. The experiment was performed in triplicate. Telomere length, measured as mean fluorescence intensity (FI), strongly correlated with telomere length measured by Southern blot analysis using the telomeric restriction fragment (TRF). The mean telomere length is expressed in kb.

### 7.8. Telomere-Centromere Staining and M-FISH Staining

Cytogenetic slides were subjected to telomere and centromere (Eurogenetec, Liege, Belgium) staining followed by M-FISH (MFISH 24XCyte, Metasystems, Altlussheim, Germany) technique. The protocol was described previously [44,52].

### 7.9. Micronucleus Assay

Micronucleus assay was performed in the absence of cytochalasineB. The protocol was previously described [65]. In addition cytochalasine B was added to analyze cell segregation in HL cell lines after four day of culture.

### 7.10. Western Blot Analysis

TRF1, TRF2 and Ku 86 expressions were assessed by western blotting on lysates of HL cell lines. Cells were sonicated in 500 µL of a buffer containing 8 M urea, 150 mM β-mercaptoethanol, 50 mM Tris-HCl (pH 7.2) and centrifuged for 30 min at 4 °C to remove cellular debris. Samples were subjected to electrophoresis on 12% (p53 and Ku) or 6% (DNA-PKcs) SDS–polyacrylamide gels, blotted onto nitrocellulose membranes, and developed using the ECL system (Amersham, Uppsala, Sweden). To verify that equivalent amounts of each sample were loaded, the filters were additionally probed with anti-actin antibody (AC74, Sigma). Densitometry was performed to evaluate the intensity of Ku70, p53, DNA-PKcs, and actin bands.

### 7.11. Apoptosis, Cell Cycles and Transcriptional Response after In Vitro Irradiation of HL Cell Lines

To evaluate the sensitivity of cell lines to apoptosis, 10^6^ cells were irradiated at 6Gy and harvested 24-h after irradiation. The protocol was previously published [31].

Cell-cycle fractions were determined by Hoechst nuclear staining. Briefly, cells were harvested, washed in PBS, and incubated in Hoechst solution for 30 min at room temperature. Data were collected using a FACSCalibur flow cytometer (BD Biosciences) and analyzed using FlowJo Version 7.5.5. The results represent the mean value of three independent experiments.

Basal and post-irradiation expression of five radiation-responsive genes (CCNG1, PUMA, P21, ZMAT3 and SESN1) was determined by quantitative real-time PCR in HL cell lines at 2 h and 24 h after 2Gy irradiation. The presented data show the rapport between basal gene expression and that after exposure. The protocol was previously published [25].

### 7.12. RNA Extraction and Transcriptome Analysis

Total RNA was extracted from frozen HL cells and controls (REMB lymphoblastoide cell line) using the AllPrep DNA/RNA/protein Mini kit (Qiagen, Hilden, Germany), quality-controlled using a 2100 BioAnalyzer (Agilent, Santa Clara, CA, USA), and quantified using a Nanodrop 2000c spectrophotometer (Thermo Scientific, Wilmington, DE, USA). The WT Expression Kit (Ambion Inc, Austin, TX, USA) was used to prepare cDNA from 10 μg purified cRNA, originally synthesised and purified from 0.25 μg of total RNA, following the manufacturer’s instructions.

### 7.13. Micronucleus and Chromosomal Aberration Scoring

Micronucleus (MN) scoring was performed after telomere and centromere staining. Automated scoring of mononuleated cells was performed using Metafer 4 image analyser software (MetaSystems, version 3.9.1) and a Zeiss Axioplan 2 imager . Ten thousand mononucleated cells per culture (two cultures per cell line, i.e., 20,000 cells per cell line) were processed in the image analyzer. Following telomere and centromere staining, MN were classified based on the detection of centromeric regions and telomeric sequences. We detected MN with telomeric and centromeric sequences (malsegregation of whole chromosomes), MN with only telomeric sequences (acentric chromosome or chromatid fragments), and MN without any sequences (interstitial deletions). In addition, nucleoplasmic bridges (NPBs) were assessed following telomere and centromere staining (dicentric chromosomes caused by telomere end fusion) or without (dicentric chromosomes resulting from misrepair of DNA strand breaks). Nuclear buds (NBUDs) were also assessed to study the process of elimination of amplified DNA possibly generated via BFB cycles (with telomere sequences or without) or the process of elimination of excess chromosomes may occur in polyploidy cells to facilitate aneuploidy rescue. 

The scoring of chromosomal aberrations was performed after telomere and centromere staining to score dicentric chromosomes and various types of acentric fragments with four telomeres (resulting from a fusion event), two telomeres, representing terminal deletions, and acentric fragments without any telomere sequence, representing interstitial deletions. Karyotype analysis was then performed using the M-FISH technique on the same slide. The introduction of telomere and centromere staining in the establishment of complex karyotypes allows easy detection and robust classification of chromosomal abnormalities. Complex chromosomal rearrangements were defined as those chromosomes involved in three or more chromosomal exchanges.

Similarly BFB chromosomes were defined as those chromosomes that were involved in multiple cycles of repetitive fusions and breakage following the loss of a telomere [66].

Metaphase images were acquired using automated acquisition module Autocapt software (MetaSystems, version 3.9.1) and a ZEISS Plan-Apochromat 63×/1.40 oil and CoolCube 1 Digital High Resolution CCD Camera. The analysis was carried out using Isis software (MetaSystems, version 5.5). Automatic scoring of MN following TC staining was performed using MN-score software (MetaSystems, version 5.5). Spectral karyotype analysis was performed using Isis software (version 3.9.1, MetaSystems, Newton, MA, USA).

### 7.14. Statistical Analysis

Linear regression, supported by the least squares method and the Fisher test, was performed to describe the relationship between the chosen factors. The normality of signal distribution was verified with the Shapiro-Wilk test. Because of the significant departure from normality, the non-parametric tests: Wilcoxon rank sum test and exact Wilcoxon-Mann-Whitney test were used to compare telomere length, telomere loss and telomere deletion in NS-HL and MC-HL patients versus healthy donors.

For transcriptome analysis, the KMH2 and L1236 cell lines (MC) were considered as one group (6 samples) and L428, L591, SUP, HDLM2 cell lines as another (NS, 12 samples). The Shapiro-Wilk test was applied to check on distribution normality. The hypotheses on the equality of median values between groups were tested by nonparametric exact Wilcoxon-Mann-Whitney test due to non-normal signal distributions. Benjamini-Hochberg procedure was used to control False Discovery Rate caused by multiple testing. 

For each transcript, the effect size (ES) translated to the Spearman’s r correlation coefficient was calculated using a formula dedicated for non-normal distribution and the U-Mann-Whitney test [67]. According to the obtained values, the transcripts were classified as of small, medium or large effect following the rules proposed by Cohen [68] (small: r ≤ 0.14; medium, 0.14 < r ≤ 0.42 and large r > 0.71). Functional analysis was performed on differentially expressed genes with at least medium effect, independently of the trend in their response.

## 8. Conclusions

Here, we provide the first evidence for the involvement of MSI to the genomic instability observed in HL. The second main finding in this study is the existence of two different mechanisms that effect chromosomal instability in HL, reflecting the clinical differences observed in terms of remission and survival of NS and MC-HL patients [69]. This study provide direct evidence that telomere dysfunction in small diploid cells in NS-HL, relative to that of HRS cells, occurred early in the transformation process, leading to the formation of dicentric chromosomes and complex chromosomal aberrations and gene amplification via B/F/B cycles. Perhaps NS-HL is associated with genetic defects in telomere replication and extension.

In the MC HL cell lines, we demonstrate that chromosomal instability is not only related to telomere dysfunction, but that dicentric formation was induced through a specific non-homologous end joining (NHEJ) pathway of DSB repair, essentially in the pericentromeric region. These processes involve the formation of specific dicentric chromosomes with both centromeres in close proximity. This may result from difference in radiation sensitivity. In line with HL cell lines findings, we report telomere dysfunction in circulating lymphocytes from NS-HL patients and high telomere heterogeneity in MC-HL patients. Altogether, our results suggest that telomere dysfunction in SN-HL induces chromosomal instability necessary for HL initiation.

## Figures and Tables

**Table 1:** The status of the five quasi-monomorphic mononucleotide repeat markers used to study Microsatellite Instability (MSI) in HL cell lines.

	Microsatellite (MSI) *					
Cell Line	PE83 NR21	PE31 BAT26	PE47 BAT25	PE85 NR24	PE84 NR2	RATIO *
L428	MSI	MSI	MSI	AIA	MSI	4/5
L591	AIA	AIA	AIA	AIA	MSI	1/5
SUP-HD1	AIA	AIA	AIA	AIA	MSI	1/5
L540	MSI	AIA	AIA	AIA	AIA	1/5
HDLM-2	MSI	MSI	MSI	AIA	AIA	3/5
L1236	AIA	AIA	AIA	AIA	AIA	0/5
KMH2	AIA	MSI	MSI	AIA	MSI	3/5

* 0/5 = stable; 4/5 = unstable.

**Table 2:** Origins, histological types, and EBV status of the HL cell lines used in this study.

Cell Line	Origin	Histology Type	EBV Status	Gender (Age)
L428	B-cell	Nodular Sclerosis (young)	−	Female (37)
L591	B-cell	Nodular Sclerosis (young)	+	Female (31)
SUP-HD1	B-cell	Nodular Sclerosis (young)	−	Male (33)
L540	T-cell	Nodular Sclerosis (young)	−	Female (20)
HDLM2	T-cell	Nodular Sclerosis (old)	−	Male (74)
L1236	B-cell	Mixed Cellularity	−	Male (34)
KMH2	B-cell	Mixed Cellularity	−	Male (37)

−: negative; +: positive.

**Table 3:** Characteristics of the HL patients.

Characteristics	No. of Patients
Nodular Sclerosis (NS)	100
Male	53
Age	33 (17–81)
Treatment	
Combined modality	100%
Mixed Cellularity (MC)	23
Male	15
Age	39 (19–69)
Treatment	
Combined modality	100%

## Supplementary Materials

The following are available online at http://www.mdpi.com/2072-6694/10/7/233/s1.
