# Erratum: Cuceu, C., et al. Independent Mechanisms Lead to Genomic Instability in Hodgkin Lymphoma: Microsatellite or Chromosomal Instability. *Cancers* 2018, *10*, 233

**DOI:** 10.3390/cancers11060757

**Published:** 2019-05-30

**Authors:** Corina Cuceu, Bruno Colicchio, Eric Jeandidier, Steffen Junker, François Plassa, Grace Shim, Justyna Mika, Monika Frenzel, Mustafa AL Jawhari, William M. Hempel, Sylwia Kabacik, Aude Lenain, Luc Morat, Theodore Girinsky, Alain Dieterlen, Joanna Polanska, Christophe Badie, Patrice Carde, Radhia M’Kacher

**Affiliations:** 1 Radiobiology and Oncology Laboratory, CEA, iRCM, 92265 Fontenay aux Roses CEDEX, France; cuceu_corina@yahoo.com (C.C.); graceshim1@gmail.com (G.S.); monika.frenzel@hotmail.com (M.F.); mustafa.aljawhari@hotmail.fr (M.A.J.); williamhempel824@gmail.com (W.M.H.); audelenain@yahoo.fr (A.L.); luc.morat@cea.fr (L.M.); 2 IRIMAS, Institut de Recherche en Informatique, Mathématiques, Automatique et Signal, Université de Haute-Alsace, 68093 Mulhouse, France; bruno.colicchio@uha.fr (B.C.); alain.dieterlen@uha.fr (A.D.); 3 Department of Genetic, Groupe Hospitalier de la Région de Mulhouse Sud-Alsace, 68093 Mulhouse, France; jeandidiere@ghrmsa.fr; 4 Institute of Biomedicine, University of Aarhus, DK-8000 Aarhus, Denmark; sjunker@biomed.au.dk; 5 Laboratory of Biochemistry B, Saint Louis Hospital, 75010 Paris, France; lfplassa@yahoo.fr; 6 Faculty of Automatic Control, Electronics and Computer Science, Silesian University of Techology, 44-100 Gliwice, Poland; justyna.mika@polsl.pl (J.M.); Joanna.Polanska@polsl.pl (J.P.); 7 Biological Effects Department, Centre for Radiation, Chemical and Environmental Hazards, Public Health England, Didcot OX11 ORQ, UK; Sylwia.Kabacik@phe.gov.uk (S.K.); christophe.badie@phe.gov.uk (C.B.); 8 Department of Radiation Oncology, Gustave Roussy Cancer Campus, 94805 Villejuif, France; theogirinsky@me.com; 9 Department of Medicine, Gustave Roussy Cancer Campus, University Paris Saclay, 94805 Villejuif, France; dr.pcarde@gmail.com; 10 Cell Environment DNA Damages R&D Oncology Section, 75020 Paris, France

The authors wish to make the following corrections to this paper [1]:

The previous 11th author, Grainne O’Brien, should be replaced with Sylwia Kabacik.

The “Authors Contributions” statement should be changed to:

**Authors Contributions:** Conceived and designed the experiments: R.M. Performed the experiments: C.C., R.M., M.F., L.M., F.P., S.K., G.S., and A.L. Analyzed the data: B.C., E.J., S.J., R.M., M.A.J., C.B., J.M., and P.C. Contributed reagents/materials/analysis tools: A.D., T.G., J.P., and P.C. Wrote the paper: R.M., W.M.H., P.C., E.J. and S.J.

The authors would like to apologize for any inconvenience caused to the readers by these changes.
