# Supplementary material for: Independent Mechanisms Lead to Genomic Instability in Hodgkin Lymphoma: Microsatellite or Chromosomal Instability
Source: Cancers (Basel). 2018 Jul 13;10(7):233. doi: 10.3390/cancers10070233 (PMC6071189; doi:10.3390/cancers10070233)
Supplement: Supplementary file 1 [file cancers-10-00233-s001.pdf]

Supplementary Materials: Independent Mechanisms Lead to Genomic Instability in Hodgkin Lymphoma: Microsatellite or Chromosomal Instability†

Corina Cuceu [1], Bruno Colicchio [2], Eric Jeandidier [3], Steffen Junker [4], François Plassa [5], Grace Shim [1], Justyna Mika [6], Monika Frenzel [1], Mustafa AL Jawhari [1], William M. Hempel [1], Grainne O'Brien [7], Aude Lenain [1], Luc Morat [1], Leonhard Heidingsfelder [8], Claire Borie [9], Dima Jouni [9], Noufissa Oudrhiri [9], Annelise Bennaceur-Griscelli [9], Lewandowski Daniel [10], Francis Finot [11], Theodore Girinsky [12], Alain Dieterlen [2], Sylvie Chevillard [13], Joanna Polanska [6], Christophe Badie [7], Patrice Carde [14] and Radhia M'Kacher [1,15,]*

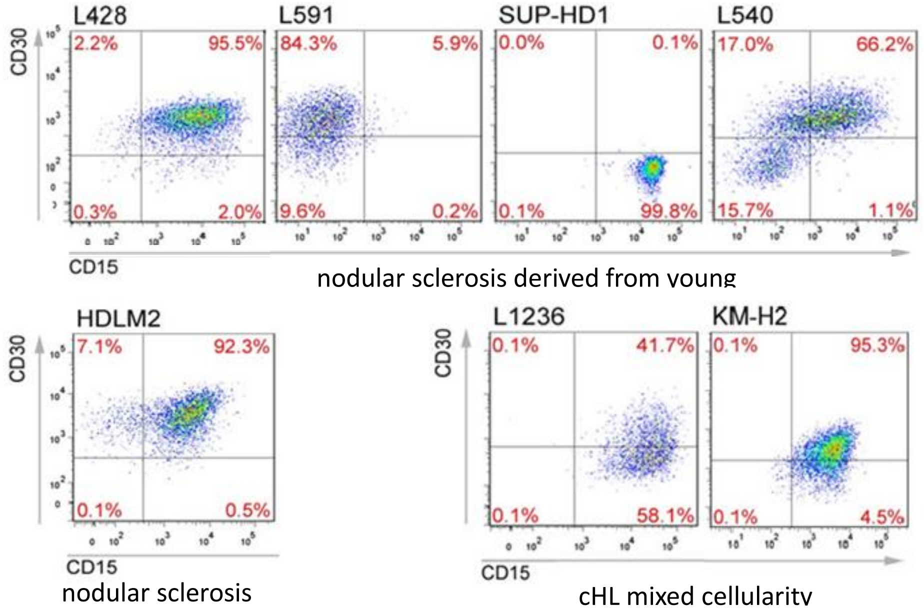

(**A**)

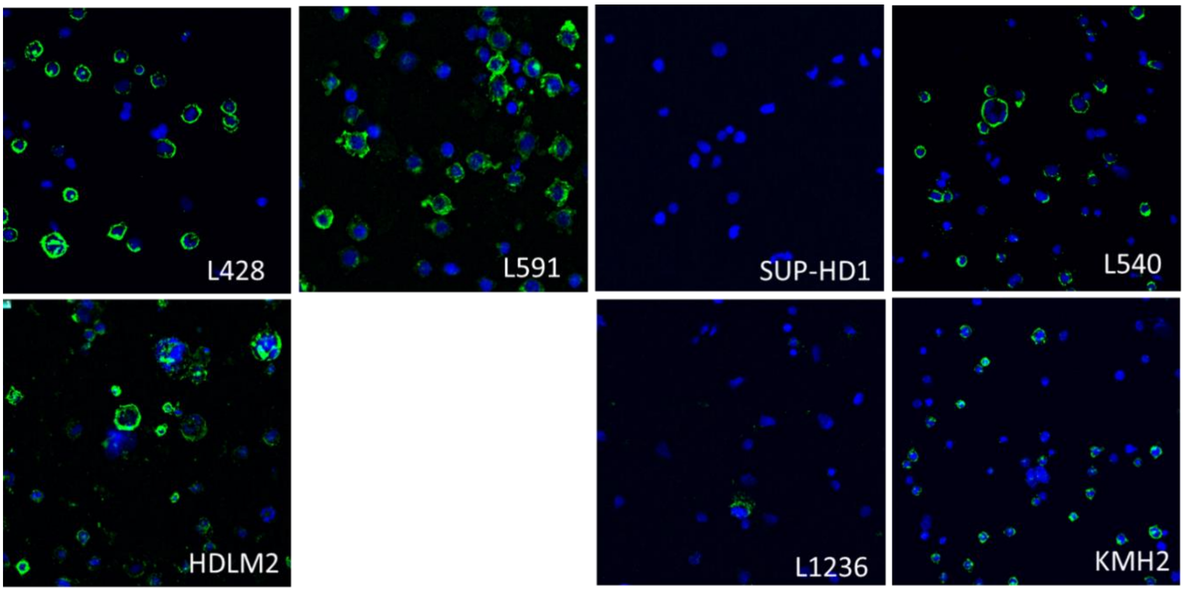

(**B**)

Figure S1. (**A**) Immunophenotype Characterization of HL cell lines. Immunophenotype characterization using CD30 and CD15, the clinical markers of HL cell lines, demonstrate the heterogeneity in the expression of these two markers. **(B)** Immunofluorescence staining with CD30 antibody (green) on HL cell lines demonstrate the high heterogeneity of CD30 expression**.**

A

Cell line	TP53				TP53 gene copy number	Copies of chromosome 17
	FASAY-RED	SPLIT 5-RED	SPLIT 3-RED	RESULTS		
L428	100	100	0	MUT	6/7 genes	6 copies
L591	3	ND	ND	WT	2 genes	2 copies
SUP-HD1	3	ND	ND	WT	ND	ND
L540	5	ND	ND	WT	3 genes	4 copies
HDLM-2	100	1	94	MUT	1 gene	2 copies
L1236	100	3	30	MUT	2/3 genes	3 copies
KM-H2	2	ND	ND	WT	2 genes	3 copies

B

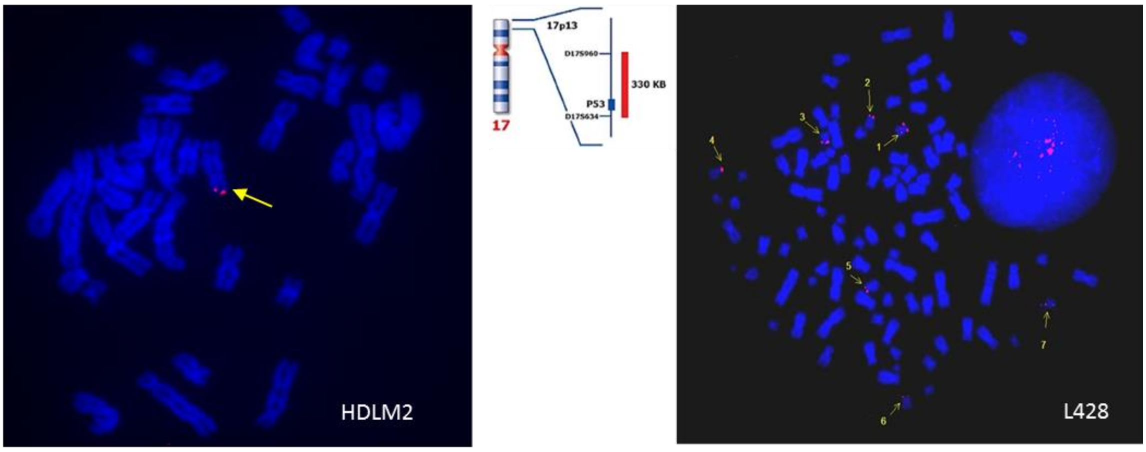

Figure S2. *TP53* status in HL cell lines, (A) *TP53* mutations were detected by a functional assay that tests the transcriptional competence of human *TP53* expressed in yeast. The copy number of the *TP53* and chromosome 17 was determined using the FISH technique. (B) illustration of *TP53* copy number (red) in the HDLM2 cell line, showing the presence of only one allele, and the L428 cell line showing the presence of a higher copy number of the *TP53* gene (red) relative to the of copy number of chromosome 17.

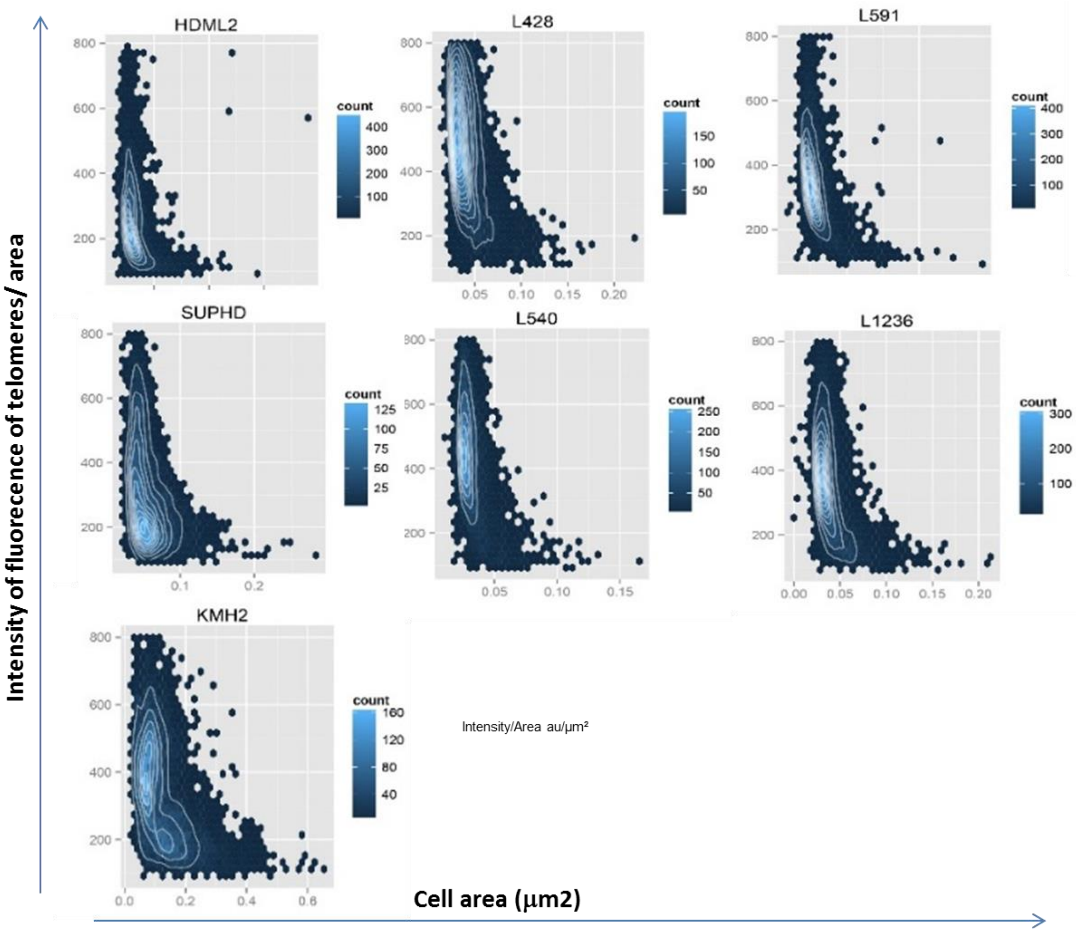

Figure S3. Intensity of fluorescence of telomeres normalized by the area of cells is represented as a function of the area of the cells. The large cells exhibited significantly lower fluorescence intensity of telomeres than small cells. The KMH2 cell line showed the presence of two populations with significantly different telomere lengths.

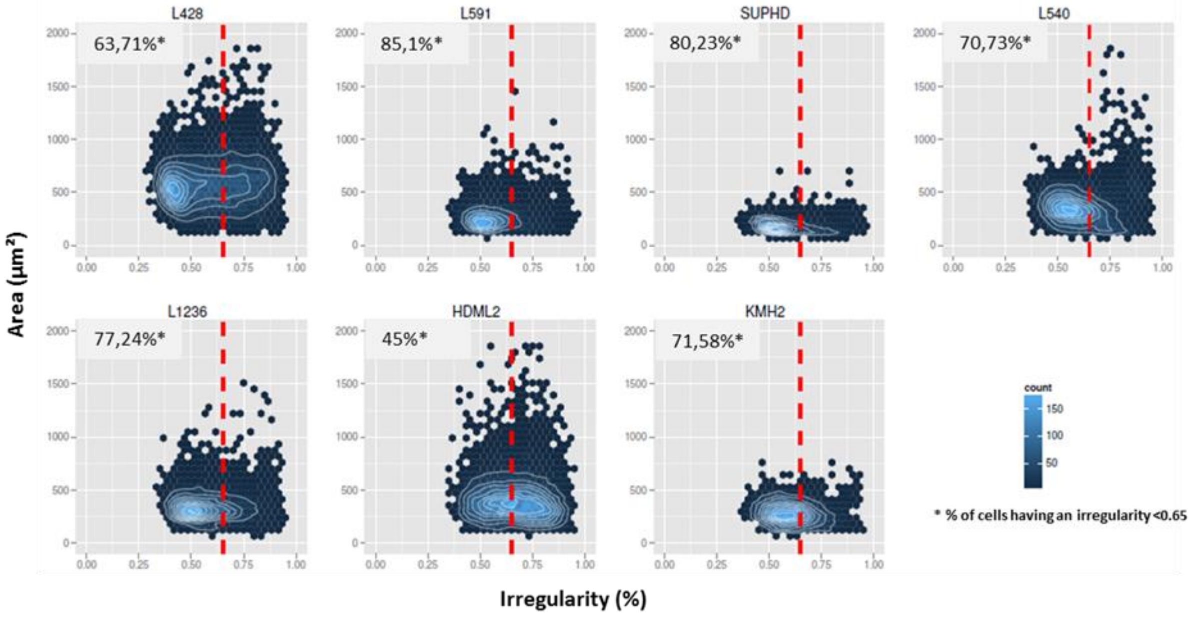

Figure S4. Nuclear irregularity was assessed using Metacyte software (Metasystem). The coefficient of perfect regularity of the nuclei (rapport between larger and longer) was equal to

1. We considered the nuclei to be irregular when the coefficient was less than 0.65. Five HL cell lines exibited more than 70% irregular nuclei.

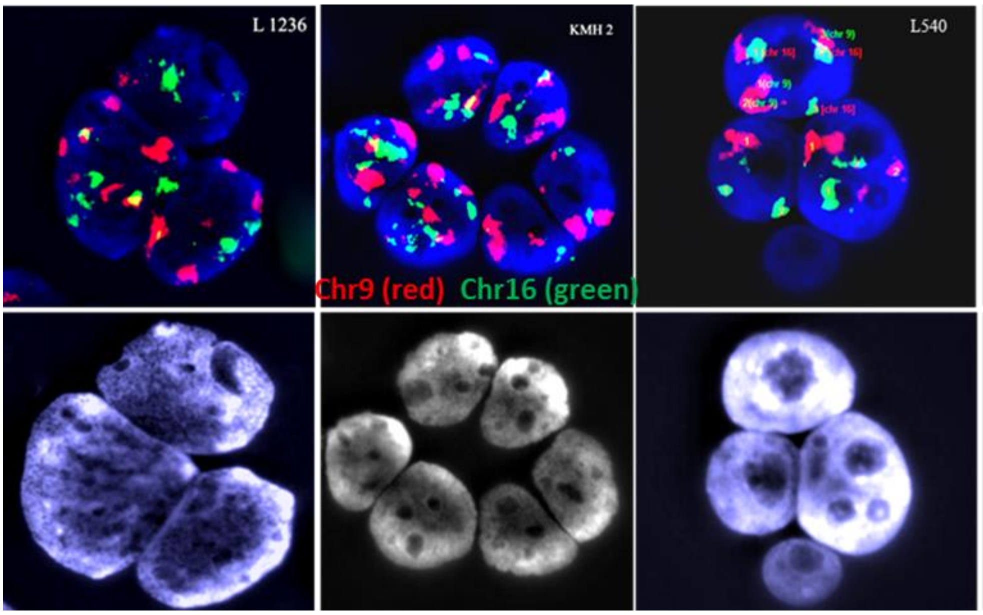

Figure S5. Chromosome 9 (red) and 16 (green) painting in L1236, KMH2, and L540 cells after four days of culture in the presence of cytochalasin B demonstrated the misegragation of these two chromosomes involving numerical chromosomal aberrations in HL.

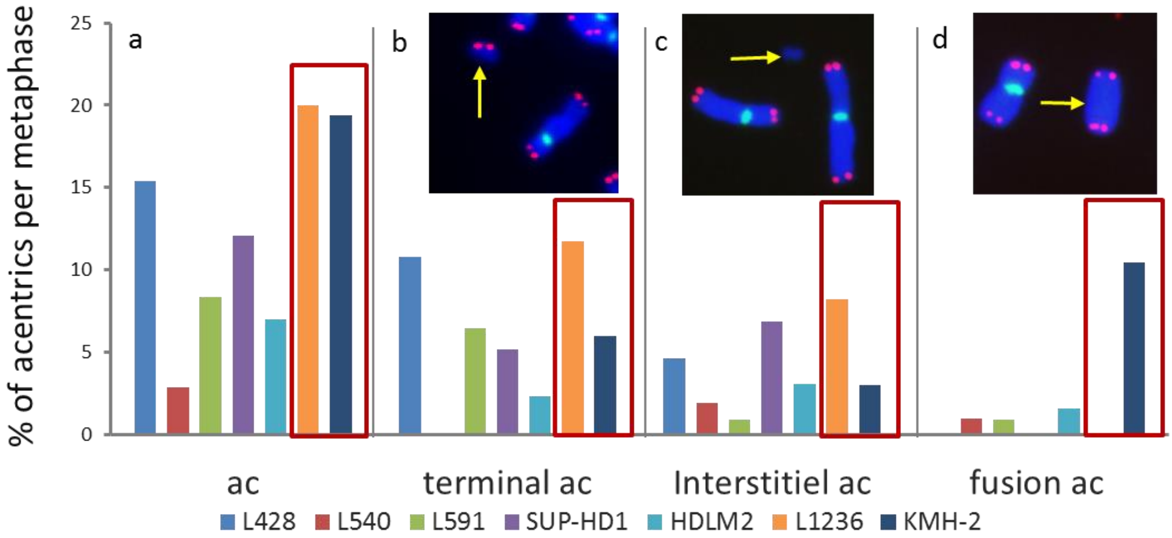

Figure S6. The frequency of acentric chromosomes detected by telomere and centromere staining in HL cell lines. (A) Frequency of acentric chromosomes. (B) Frequency of terminal deletions (with only two telomeres). (C) Frequency of interstitial acentric chromosomes (without any staining). (D) Frequency of acentric chromosomes resulting from the fusion of two terminal deletions (with four telomeres).

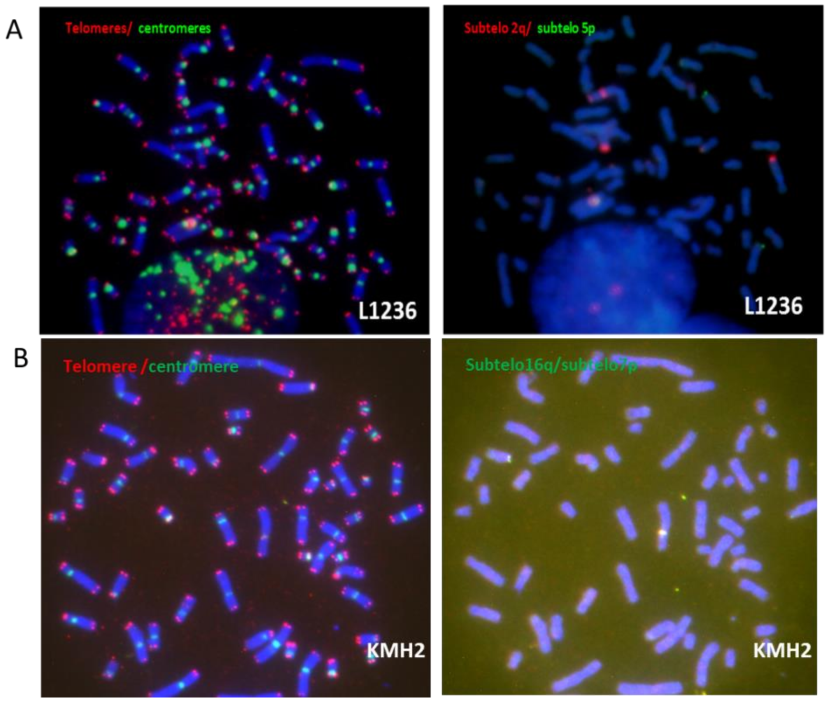

Figure S7. (A) Sequential analysis using telomere and centromere staining followed by subtelomere of chromosome2q (red) and 5p (green) painting in L1236 cells revealed the absence of subtelomere sequences in dicentric chromosome breakpoints. **(B)** Similarly, the absence of subtelomere 16p and 7p in dicentric chromosomes were observed in addition to the loss of the subtelomeric region. Only one copy was detected.

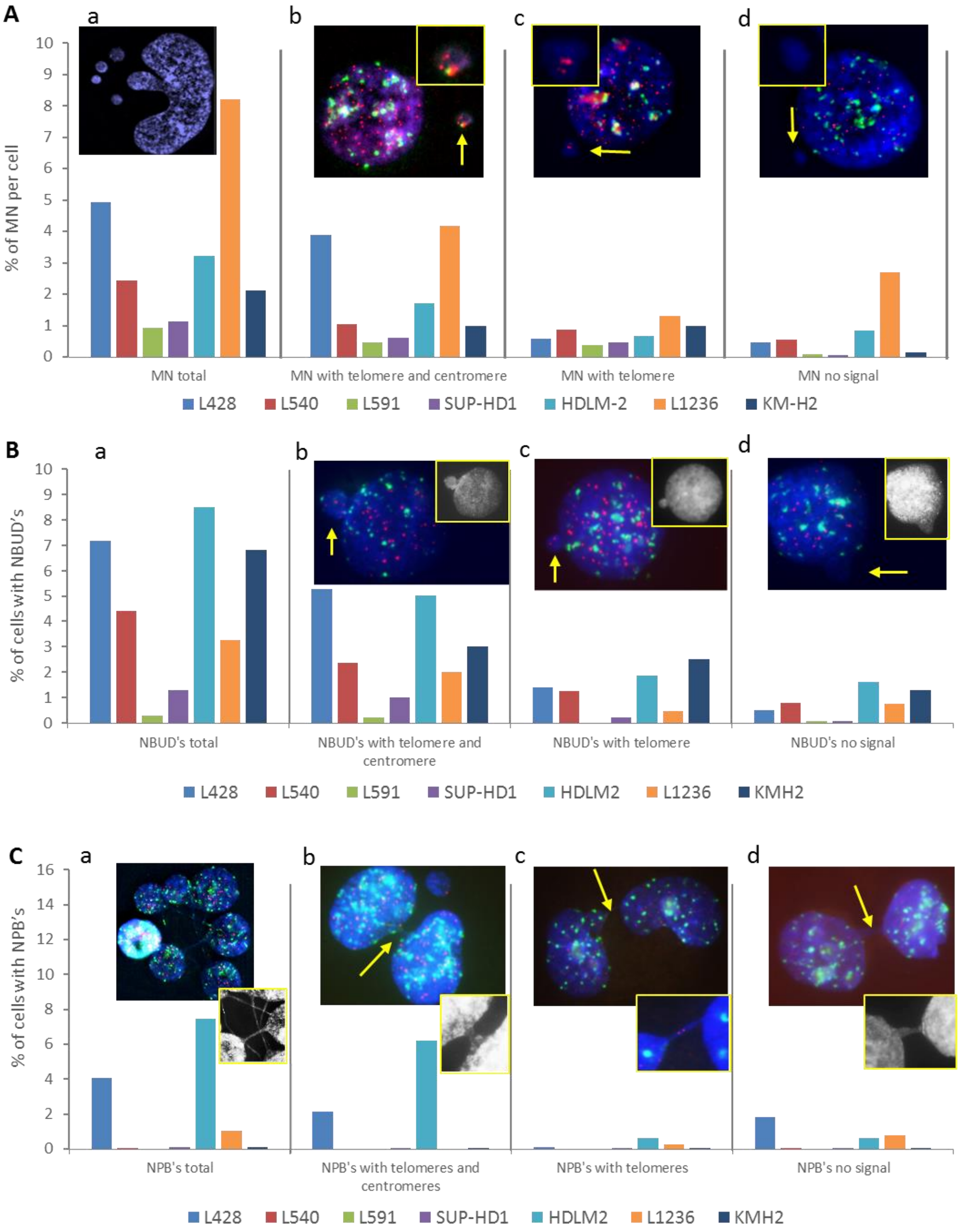

Figure S8. The frequency of micronucleus (MN), nuclear buds (NBUDs) and nucleoplasmic bridges (NPBs) assessed following telomere and centromere staining (Aa) the frequency of MN after homogeneous staining (DAPI) (Ab)the MN with telomere and centromere staining (Ac) MN with only telomere sequences (Ad) MN without any staining.(Ba) The frequency of NBUDs after homogenous staining (Bb) the frequency of NBUDs with telomere and centromere staining (Bc) NBUDs with telomere staining (Bd) NBUDs without telomere and centromere staining.(Ca) the frequency of NPBs after homogenous staining (Cb) NPBs with centromere and telomere staining (Cc) NPBs with only centromere staining (Cd) NPBs without any staining.

Table S1. Clonal chromosomal aberrations detected after telomere and centromere staining followed by M-FISH technique in HL cell lines. Thirty metaphases were analyzed in each cell line.

Cell lines	Chr Number	Clonal aberrations
L428	94 (71-103)	dic(X;21)[29],der(1)t(X;1)[29],2xder(2)t(2;8)[29],dic(3;15)[30],dic(5;13)[29], der(5)t(5;6)[23],i(5)(q),del(6p),der(7)t(7;15)[29],der(8)t(8;18)[2],der(9)t(9;11;21)[16], der(9)t(9;14)[22],der(12)t(7;12)[28],der(14)t(14;9)[16],der(16)t(16;9;16;9;1)[30], der(14)t(14;17)[16],del(12p),i(12)(p10),del(17p),der(17)t(2;17)[23],der(18)t(7;18)[12]
**L591**	45 (29-49)	der(2)t(2;8)[29],der(8)(8;11)[30],der(14)t(14;9)[30],der(12)t(7;12)[5]
**SUP-HD1**	41 (10-95)	amp(1)[29],der(2)t(2;8)[29],der(2)t(2;8;2)[26],der(4)t(4;16)[26],der(4)t(4;18)[27], der(4)t(11;4;16)[26],del(5p),i(5)(p10),der(6)t(6;6;9)[28],2xt(8;18)[25],der(9)t(9;8;12)[30] ,der(9)t(9;12)[25],der(12)t(12;9)[21],i(14)(p10),der(15)t(2;15)[30],der(15)t(5;15)[30], der(22)t(22;13)[30],der(18)t(8;18;8)[19]
**L540**	64 (33-118)	der(X)t(X;2)[30],der(1)t(1;15)[29],der(1)t(1;5)[29],der(1)t(1;12)[30],der(2)t(2;12)[30], der(2)t(2;8),der(5)t(5;1;5)[29],der(7)t(7;19)[30],der(8)t(8;20;6)[30],der(14)t(11;14)[29], der(16)t(16;18)[30],der(16)t(X;16)[29],del(18),der(21)t(21;20)[30], der(21)t(21;17;15)[30],der(22)t(22;11;14)[30]
**HDLM-2**	36 (24-78)	der(2)t(2;8)[29],der(2)t(19;2;4;2;4)[27],der(3)t(3;4;15;14)[27],der(6)t(6;3)[29], der(7)t(7;3)[28],der(8)t(8;2)[30],der(8)t(2;8;12)[30],der(10)t(10;12)(15), der(11)t(11;16)[20],der(13)t(13;22)[29],der(13)t(13;16;11)[29],der(14)t(14;21)[28], der(14)t(14;15;6)[29],der(15)t(8;15;17;1)[25],dic(5;17)[29],dic(19;1;5;1)[28], dic(3;19;9)[25],tric(17;19;9;19)[28],tric(4;5;4)[29]
**KMH2**	57 (45-90)	der(X)t(2;4;X;4;2)[27],der(X)t(X;5)[26],der(X)t(6;X;4;2)[27],dic(X;4;2),dic(X;2;16), dic(1;7),dic(6;2;16;X),der(1)t(1;16)[30],der(2)t(2;13)[30],der(2)t(11;2;9)[30], der(3)t(3;8)[28],der(16)t(15;16;15)[30],der(4)t(4;16)[30],der(4)t(11;12)[30], der(4)t(4;12)[20],der(5)t(3;5;11)[30],der(5)t(5;1)[24],dic(7;7),der(7)t(7;9;1)[30], der(8)t(8;1;19)[30],der(8)t(1;8;3)[30],der(8)t(8;12)[22],dic(10;22;16), der(10)t(10;16;10;16)[30],der(10)t(1;10)[30],dic(10;3;15;19;12;11),dic(11;7), der(13)t(13;21)[24],der(13)t(12;13;21)[8],der(15)t(15;9;1)[30],der(15)t(5;15;7)[8], der(15)t(11,15;7)[27],der(16)t(2;8;16;21;3;7)[30],der(16)t(15;16;18)[3],der(17)t(X;17)[30] ,der(18)t(15;18)[30],der(18)t(13;18)[8],der(18)t(18;20)[19],dic(19;19), der(21)t(12;21)[30],der(22)t(22;16;3;10)[30]
**L1236**	61 (33-87)	der(1)t(1;9)[3],2xt(1;14)[26],der(1)t(1;8)[14],der(1)t(1;18)[27],dic(2;21),der(3)t(3;16)[26] ,der(4)t(4;8)[29],der(6)t(1;6)[16],der(6)t(6;9;6;8)[26],der(7)t(7;17)[25], der(7)t(17;7;18)[10],der(8)t(8;1;14)[24],dic(9;16),der(9)t(3;9)[13], der(10)t(10;14;16;19)[25],der(11)t(11;21)[27],der(12)t(12;19)[12],dic(12;17;Y),dic(12;12) ,der(15)t(15;7)[6],der(18)t(1;18)[24],der(20)t(15;20)[28],(16;19)[3],der(22)t(22;15)[25], der(22)t(22;6)[28],dic(21;t(7;17))

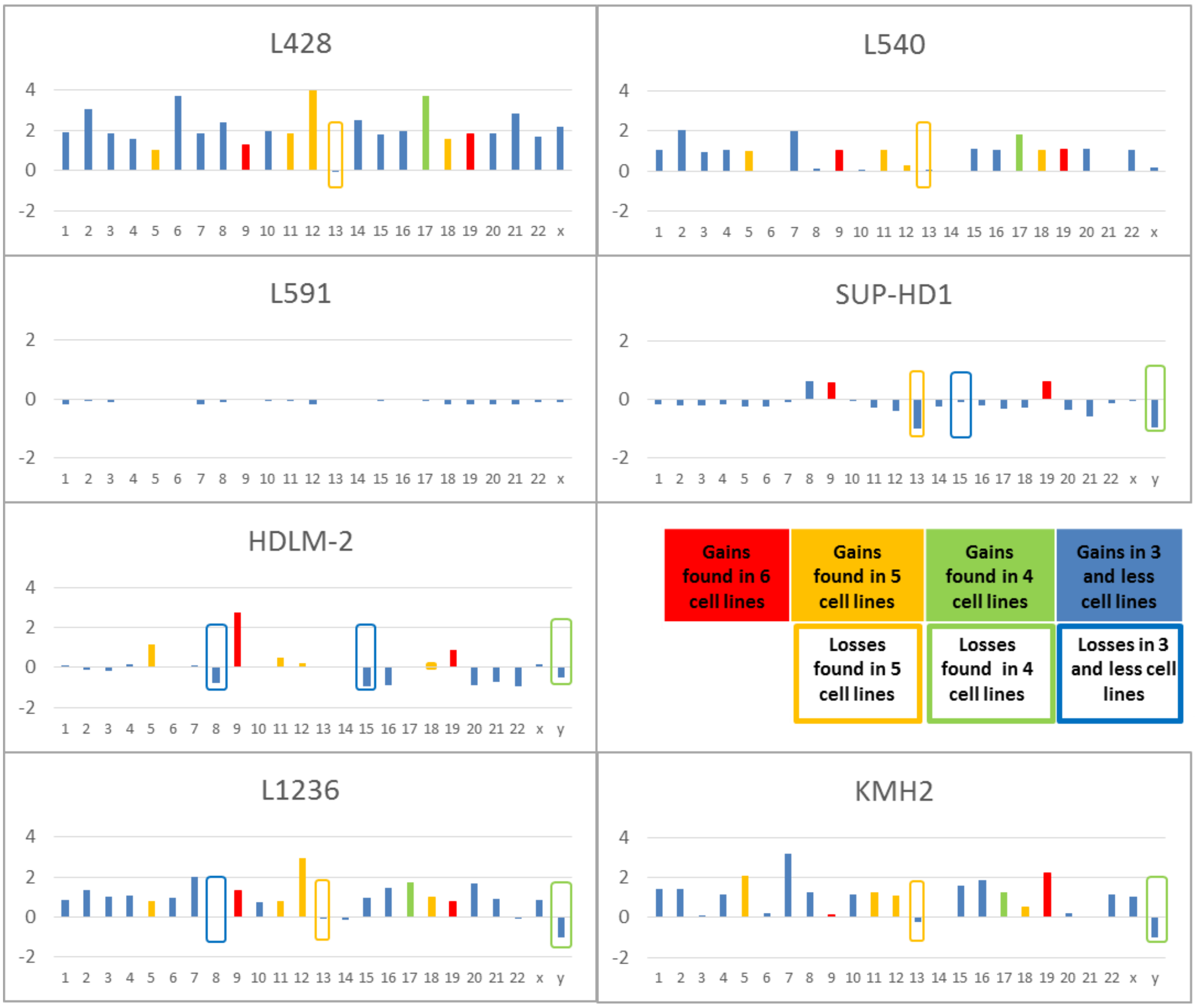

Figure S9. Numerical chromosomal aberrations including gain and loss of whole chromosomes. Gains of chromosomes 9 and 19 (marked in red) were found in 6/7 cell lines, chromosomes 5,11,12, and 18 (marked in yellow) in 5/7 cell lines, and chromosome 17 (marked in green) in 4/7 cell lines. Gains of other chromosomes was observed in at least one cell line. The loss of chromosome 13 (framed in yellow) was found in 5/7 cell lines; chromosome y (framed in green) in 4/4 cell lines, and chromosomes 8 and 15 (framed in blue) in two cell lines. Loss or gains of more copies of the same chromosome are shown on the y axis, with a value of 0 representing the normal number of chromosomes.

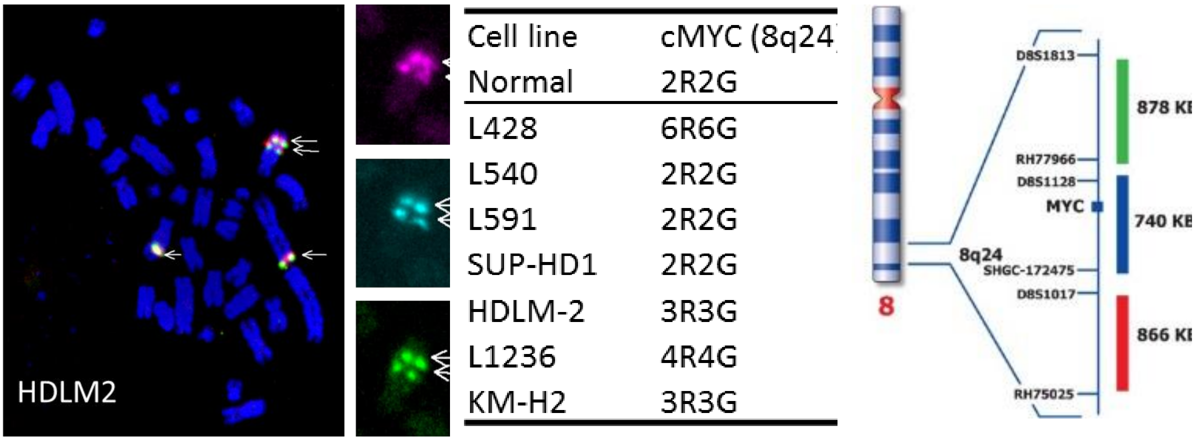

(**A**)

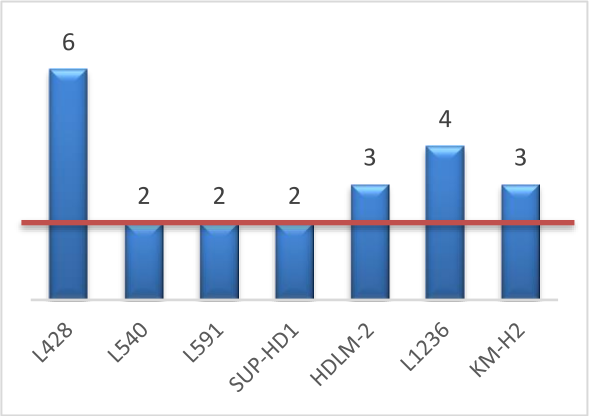

(**B**)

Figure S10. (**A**): *c-MYC* in HL cell lines was assessed using FISH. The amplification in the region containing *c-MYC* was found in L428 (6 copies), L1236 (4 copies), HDLM2 and KM-H2 (3 copies), without the mutation in *c-MYC*. (**B**): FISH with *JAK2 probe-specific* in HL cell lines. High copy numbers of the gene were found in in 6/7 cell lines; without mutation in *JAK2*.

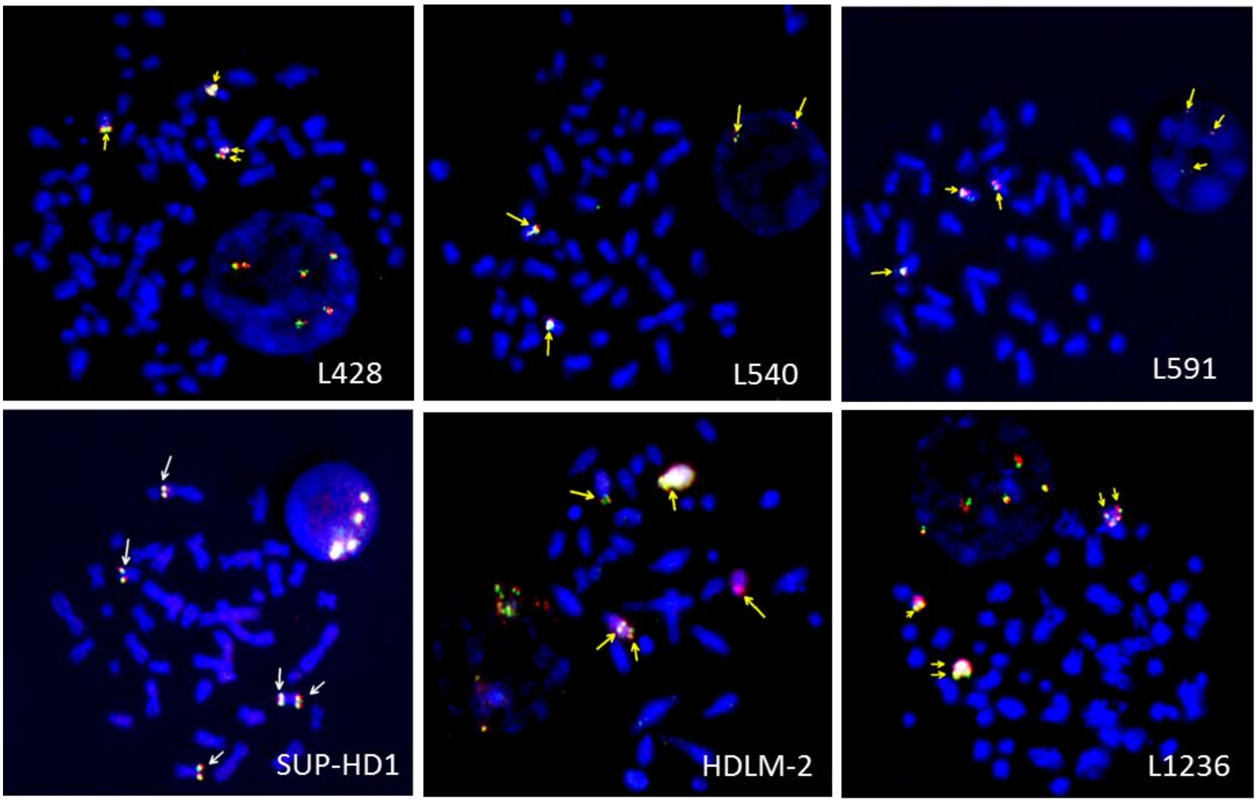

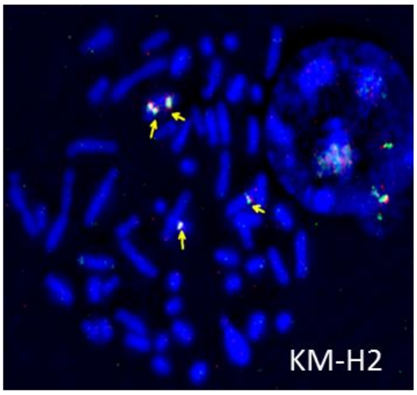

Cell line	JAK2 (9p24)
Normal	2R2G
L428	4R4G
L540	2R2G
L591	3R3G
SUP-HD1	5R5G
HDLM-2	8R7G
L1236	5R5G
KM-H2	4R4G

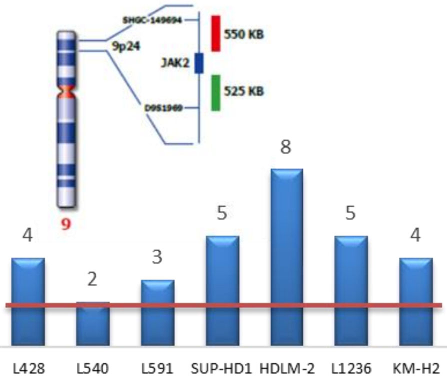

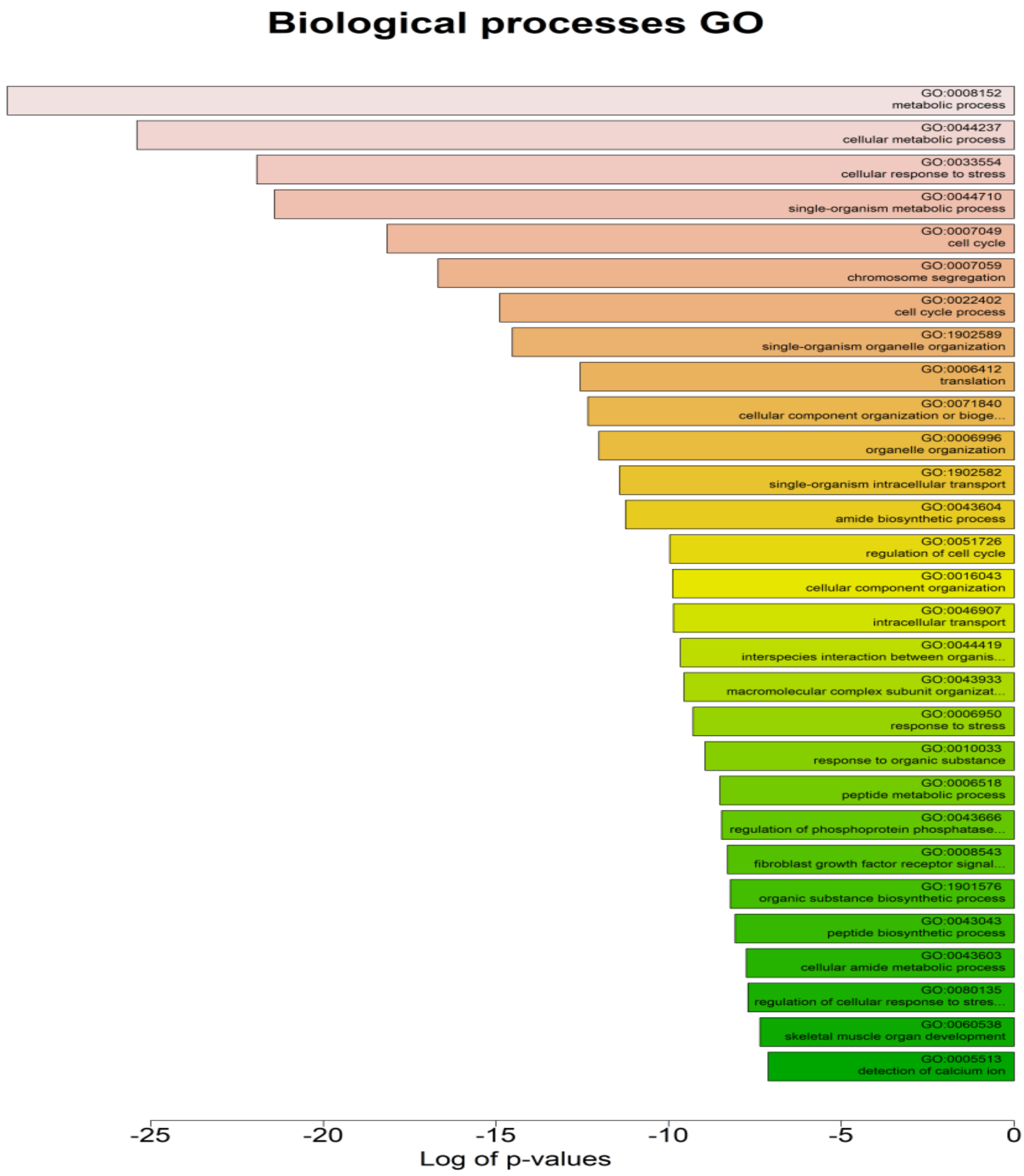

Figure S11. Transcriptome analysis revealed the affected of biological process.

Table S2. Significant difference was observed in the expression of 140 DNA repair transcripts (78 unique genes) between NS-HL and MC-HL cell lines. Adjusted p-value and effect size were calculated. If effect size value is negative, that means that the transcript was down-regulated in SN. If the effect-size value is positive, then NS was up-regulated.

Gene_id	Symbol	TRANSCRIPTS.ID	Gene.Name	p.vals	p_adjust.BH.	EFFECTSIZE
328	APEX1	AF071798	REV3L	0,00011	0,00345	1
983	CDK1	AF176039	HMGA1	0,00011	0,00345	-1
1032	CDKN2D	AF230406	PML	0,00011	0,00345	-1
2067	ERCC1	AK001980	PARP2	0,00011	0,00345	1
2177	FANCD2	AK055786	XRCC6	0,00011	0,00345	1
2475	MTOR	AL096744	REV3L	0,00011	0,00345	1
2547	XRCC6	AL833126	C10orf6	0,00011	0,00345	1
2956	MSH6	BC003535	C19orf40	0,00011	0,00345	1
2965	GTF2H1	BC004281	TFPT	0,00011	0,00345	1

2966	GTF2H2	BC008343	XRCC6	0,00011	0,00345	1
3146	HMGB1	BC009507	G1P2	0,00011	0,00345	-1
3159	HMGA1	BC010170	C19orf40	0,00011	0,00345	1
4331	MNAT1	BC015742	POLH	0,00011	0,00345	1
4361	MRE11A	BC018259	XRCC6	0,00011	0,00345	1
4862	NPAS2	BC020892	C10orf78	0,00011	0,00345	1
4968	OGG1	BC026066	ASCC3	0,00011	0,00345	1
5371	PML	BC034641	ESCO2	0,00011	0,00345	1
5429	POLH	BC063434	HMGA1	0,00011	0,00345	-1
5531	PPP4C	BC063458	MRE11A	0,00011	0,00345	1
5884	RAD17	BC071863	HMGA1	0,00011	0,00345	-1
5932	RBBP8	BC072460	TCEA1	0,00011	0,00345	1
5980	REV3L	BC101533	AXIN2	0,00011	0,00345	-1
5983	RFC3	BC101834	PTTG1	0,00011	0,00345	1
6233	RPS27A	BC107750	CDC2	0,00011	0,00345	1
6917	TCEA1	BC108908	POLD3	0,00011	0,00345	1
8313	AXIN2	CR456492	XRCC6	0,00011	0,00345	1
8451	CUL4A	NM_001469	XRCC6	0,00011	0,00345	1
9112	MTA1	NM_001641	APEX1	0,00011	0,00345	1
9126	SMC3	NM_001786	CDC2	0,00011	0,00345	1
9232	PTTG1	NM_001983	ERCC1	0,00011	0,00345	1
9636	ISG15	NM_002131	HMGA1	0,00011	0,00345	-1
9643	MORF4L2	NM_002912	REV3L	0,00011	0,00345	1
9978	RBX1	NM_004219	PTTG1	0,00011	0,00345	1
10038	PARP2	NM_004655	AXIN2	0,00011	0,00345	-1
10075	HUWE1	NM_005101	G1P2	0,00011	0,00345	-1
10213	PSMD14	NM_005484	PARP2	0,00011	0,00345	1
10714	POLD3	NM_006502	POLH	0,00011	0,00345	1
10973	ASCC3	NM_006756	TCEA1	0,00011	0,00345	1
11144	DMC1	NM_014248	RBX1	0,00011	0,00345	1
11200	CHEK2	NM_031407	HUWE1	0,00011	0,00345	-1
11277	TREX1	NM_033379	CDC2	0,00011	0,00345	1

Table S2. *Cont.*

22992	KDM2A	NM_052865	C20orf72	0,00011	0,00345	1
25788	RAD54B	NM_080648	APEX1	0,00011	0,00345	1
27030	MLH3	NM_080649	APEX1	0,00011	0,00345	1
27343	POLL	NM_138376	TTC5	0,00011	0,00345	1
29844	TFPT	NM_145247	C10orf78	0,00011	0,00345	1
50484	RRM2B	NM_152266	C19orf40	0,00011	0,00345	1
54617	INO80	BC031215	NUDT16	0,00022	0,00564	0,972
54840	APTX	M73778	PML	0,00022	0,00564	-0,972
55031	USP47	NM_001002759	C10orf78	0,00022	0,00564	0,972
55719	SLF2	NM_004958	FRAP1	0,00022	0,00564	-0,972
55775	TDP1	NM_005590	MRE11A	0,00022	0,00564	0,972
56946	EMSY	NM_005805	PSMD14	0,00022	0,00564	0,972
56949	XAB2	NM_006591	POLD3	0,00022	0,00564	0,972

57332	CBX8	NM_006828	ASCC3	0,00022	0,00564	0,972
57697	FANCM	NM_007068	DMC1	0,00022	0,00564	0,972
58493	INIP	NM_012308	FBXL11	0,00022	0,00564	0,972
79008	SLX1B	NM_033246	PML	0,00022	0,00564	-0,972
80198	MUS81	NM_033249	PML	0,00022	0,00564	-0,972
91442	FAAP24	NM_079421	CDKN2D	0,00022	0,00564	0,972
91875	TTC5	XM_495886	RNF169	0,00022	0,00564	0,972
92667	MGME1	AK021600	POLL	0,00043	0,00912	0,944
119392	SFR1	BC000803	GIYD2	0,00043	0,00912	-0,944
131870	NUDT16	BC030565	C10orf6	0,00043	0,00912	0,944
157570	ESCO2	BC032304	RAD17	0,00043	0,00912	0,944
220064	ORAOV1	BC035658	DMC1	0,00043	0,00912	0,944
254225	RNF169	M79462	PML	0,00043	0,00912	-0,944
257218	SHPRH	NM_001005735	CHEK2	0,00043	0,00912	0,944
286053	NSMCE2	NM_001515	GTF2H2	0,00043	0,00912	0,944
404672	GTF2H5	NM_002675	PML	0,00043	0,00912	-0,944
		NM_002894	RBBP8	0,00043	0,00912	0,944
		NM_005445	CSPG6	0,00043	0,00912	0,944
		NM_013274	POLL	0,00043	0,00912	0,944
		NM_017553	INOC1	0,00043	0,00912	0,944
		NM_021218	C9orf80	0,00043	0,00912	0,944
		NM_153451	ORAOV1	0,00043	0,00912	0,944
		AB039667	MLH3	0,00075	0,0136	0,917
		AB166669	N/A	0,00075	0,0136	0,917
		AY551303	CHEK2	0,00075	0,0136	0,917
		NM_016821	OGG1	0,00075	0,0136	0,917
		NM_016828	OGG1	0,00075	0,0136	0,917
		NM_017692	APTX	0,00075	0,0136	0,917
		NM_025128	MUS81	0,00075	0,0136	0,917
		AB069681	ERCC1	0,00129	0,02031	0,889
		BC033404	C11orf30	0,00129	0,02031	0,889
		BC047324	CSPG6	0,00129	0,02031	0,889

Table S2. *Cont.*

		BC104881	APTX	0,00129	0,02031	0,889
		BX538161	APTX	0,00129	0,02031	0,889
		NM_001800	CDKN2D	0,00129	0,02031	0,889
		NM_002720	PPP4C	0,00129	0,02031	-0,889
		NM_002873	RAD17	0,00129	0,02031	0,889
		NM_006550	RAD54B	0,00129	0,02031	0,889
		NM_012286	MORF4L2	0,00129	0,02031	-0,889
		NM_020937	FANCM	0,00129	0,02031	0,889
		NM_145862	CHEK2	0,00129	0,02031	0,889
		NM_207118	GTF2H5	0,00129	0,02031	0,889
		AB163437	RRM2B	0,00205	0,02824	0,861
		AB166670	RRM2B	0,00205	0,02824	0,861
		AY551298	CHEK2	0,00205	0,02824	0,861

	BC112167	MLH3	0,00205	0,02824	0,861
	NM_002431	MNAT1	0,00205	0,02824	0,861
	NM_002954	RPS27A	0,00205	0,02824	-0,861
	NM_004689	MTA1	0,00205	0,02824	-0,861
	NM_016381	TREX1	0,00205	0,02824	-0,861
	NM_020196	XAB2	0,00205	0,02824	-0,861
	NM_173685	C8orf36	0,00205	0,02824	0,861
	AF508978	MTA1	0,00323	0,03878	-0,833
	AK127264	USP47	0,00323	0,03878	0,833
	AY208830	APTX	0,00323	0,03878	0,833
	AY208833	APTX	0,00323	0,03878	0,833
	BC000149	RFC3	0,00323	0,03878	0,833
	BC008930	ERCC1	0,00323	0,03878	0,833
	BC072383	NPAS2	0,00323	0,03878	-0,833
	BC108261	RRM2B	0,00323	0,03878	0,833
	BX648322	SHPRH	0,00323	0,03878	0,833
	NM_001008744	TDP1	0,00323	0,03878	-0,833
	NM_001008895	CUL4A	0,00323	0,03878	0,833
	NM_001018115	FANCD2	0,00323	0,03878	0,833
	NM_002128	HMGB1	0,00323	0,03878	0,833
	NM_002915	RFC3	0,00323	0,03878	0,833
	NM_005316	GTF2H1	0,00323	0,03878	0,833
	NM_013342	TFPT	0,00323	0,03878	0,833
	NM_016819	OGG1	0,00323	0,03878	0,833
	NM_020649	CBX8	0,00323	0,03878	-0,833
	NM_033239	PML	0,00323	0,03878	-0,833
	NM_203292	RBBP8	0,00323	0,03878	0,833
	Y16676	MSH6	0,00323	0,03878	-0,833

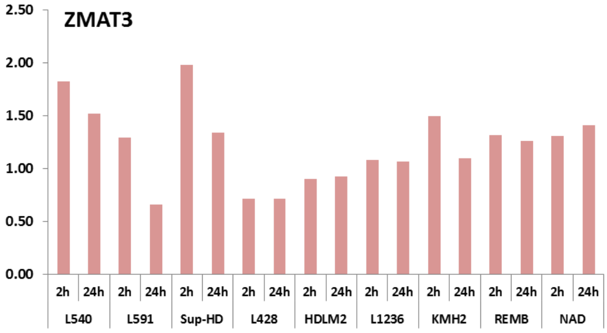

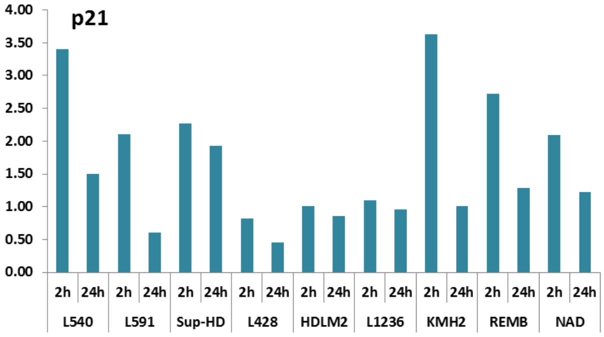

Figure S12. ZMAT3 and p21 genes expressions were analyzed 2 and 24 h after 2 Gy of irradiation by quantitative RT-PCR. Values are normalized to HPRT expression levels. The gene expression ratio of irradiated/mock-treated cells is presented. The mean values of triplicate experiments, each with three RT-PCR reactions, are shown.
